# Supplementary material for: A novel Thermodesulfovibrionia lineage largely supports sulfate reduction in sandy sediments of the southern Baltic Sea
Source: ISME Commun. 2026 Sep 4;6(1):ycag255. doi: 10.1093/ismeco/ycag255 (PMC13657043; doi:10.1093/ismeco/ycag255)
Supplement: Supplementary file 1 [file supplementary_material_ycag255.zip › MGF_SR_supplement_rev2.pdf]

Supplement

A novel Thermodesulfovibrionia lineage largely supports sulfate reduction in sandy sediments of the southern Baltic Sea

Judith Piontek[1*], Thomas Schott[1], Patricia Roeser[1,2], Jens Kallmeyer[3], Michael Ernst Böttcher[1,4], Christiane Hassenrück[1], Klaus Jürgens[1]

[1] Leibniz Institute for Baltic Sea Research Warnemünde, Seestr. 15, 18119 Rostock, Germany

[2] Environmental Geology, Institute for Geosciences, University of Bonn, Kirschallee 1-3, 53115 Bonn, Germany

[3] GFZ Helmholtz Centre for Geosciences, Telegrafenberg, 14473 Potsdam, Germany

[4] Marine Geochemistry, University of Greifswald, Friedrich-Ludwig-Jahnstr., 17489 Greifswald, Germany

*correspondence to: Judith Piontek (judith.piontek@io-warnemuende.de)

Supplementary Methods

Calculation of sulfate reduction rates

Sulfate reduction rates (SRR) were calculated by

$$\mathrm{SRR} = \frac{SO_4 * \varphi * a_{tris} * 1.06 * 10^6}{a_{tot} * t}$$

with SO_4: sulfate concentration in the pore water [mmol L^{-1}]; φ: porosity [mL cm^{-3}] of the sediment, set to 0.7; a_{tris}: radioactivity of total reduced inorganic sulfur [cpm]; a_{tot}: total radioactivity used in the spiking [cpm]; t: incubation time [d]; 1.06: correction factor for isotopic fractionation; 10^6: unit conversion factor from [µmol L^{-1}] to [pmol L^{-1}] [1].

Phylogenetic placement of dsrA sequences

For phylogenetic placement, a set of known reference sequences is used and evolutionary history is taken into account [2]. In order to examine dsrA sequences from the sediments that could not be classified beyond phylum level by similarity searches, a phylogenetic tree was constructed from a published alignment of almost 1000 taxonomically assigned *dsrA* gene sequences using Fasttree2 (v2.1.11) [3–5]. Sequences from Fehmarn Belt (FB) and Odra Bank (ODB) were added to the alignment using MAFFT (v7.525) [6]. RAxML-NG (v1.2.2) was used to evaluate the resulting ML tree and optimize all model parameters and branch lengths [7]. Subsequently, phylogenetic placement was performed using EPA-NG (v0.3.8) [8].

Phylogenomic marker gene tree

Eight *Thermodesulfovibrionia* metagenome-assembled genomes (MAGs) recovered from the Odra Bank study site were available from a previous sampling campaign. To investigate phylogenetic relationships, we compiled an additional 142 publicly available *Thermodesulfovibrionia* MAGs originating from diverse aquatic environments, together with 281 genomes assigned to the class *Thermodesulfovibrionia* in GTDB (r232). All MAGs included in the analysis met quality thresholds of >60% completeness and <3% contamination, as assessed using CheckM [9]. The resulting genome collection was used to construct a phylogenomic tree based on single-copy marker genes implemented in GTDB-Tk denovo_wf (v2.1.0), employing the LG protein substitution model with gamma-distributed rate variation [10]. The phylogenomic tree was manually collapsed according to the GTDB taxonomy [10] and visualized using iTOL v6.8.1 [11]. In addition, relative evolutionary divergence (RED) values were calculated using the get_reds function from the R package castor [12].

Statistical analysis of transcriptomic data

Compositional differences between KEGG-annotated transcriptomes of *Desulfobacterales*, *Desulfobulbales* and *Thermodesulfovibrionia* were explored by a PERMANOVA of Bray-Curtis dissimilarities using the function adonis2 in the R package vegan [11]. The marginal effects of the terms area (FB vs. ODB) and taxonomy (*Desulfobacterales*, *Desulfobulbales*, *Thermodesulfovibrionia*) were assessed (by="terms").

The R package ALDEx2 was used to identify KEGG orthologs (KOs) in the *Desulfobacterales* and *Desulfbulbales* transcriptomes that show differential expression levels in FB and ODB samples. ALDEx2 accounts for the compositional nature of sequencing data by applying a centered log-ratio transformation to Monte Carlo instances generated from the Dirichlet distribution of count data [12, 13]. Robustly differentiated KOs were identified based on effect magnitude (|diff.btw| > 1) and low overlap of Monte Carlo-estimated group distributions (overlap < 0.25).

References

1. Jørgensen BB, Fenchel T. The sulfur cycle of a coastal marine sediment. *Mar Biol* 1974;**24**:189–201.
2. Czech L et al. Metagenomic Analysis Using Phylogenetic Placement—A Review of the First Decade. *Front Bioinforma* 2022;**2**:1–25. https://doi.org/10.3389/fbinf.2022.871393
3. Diao M et al. Global diversity and inferred ecophysiology of microorganisms with the potential for dissimilatory sulfate/sulfite reduction. *FEMS Microbiol Rev* 2023;**47**:1–18. https://doi.org/10.1093/femsre/fuad058
4. Price MN, Dehal PS, Arkin AP. FastTree 2 - Approximately maximum-likelihood trees for large alignments. *PLoS One* 2010;**5**. https://doi.org/10.1371/journal.pone.0009490
5. Pelikan C et al. Glacial Runoff Promotes Deep Burial of Sulfur Cycling-Associated Microorganisms in Marine Sediments. *Front Microbiol* 2019;**10**:1–17. https://doi.org/10.3389/fmicb.2019.02558
6. Katoh K et al. MAFFT: A novel method for rapid multiple sequence alignment based on fast Fourier transform. *Nucleic Acids Res* 2002;**30**:3059–3066.

https://doi.org/10.1093/nar/gkf436

7. Kozlov AM et al. RAxML-NG: A fast, scalable and user-friendly tool for maximum likelihood phylogenetic inference. *Bioinformatics* 2019;**35**:4453–4455. https://doi.org/10.1093/bioinformatics/btz305

8. Barbera P et al. EPA-ng: Massively Parallel Evolutionary Placement of Genetic Sequences. *Syst Biol* 2019;**68**:365–369. https://doi.org/10.1093/sysbio/syy054

9. Parks DH et al. CheckM: assessing the quality of microbial genomes recovered from isolates, single cells, and metagenomes. *Genome Res* 2015;1043–1055. https://doi.org/10.1101/gr.186072.114

10. Chaumeil PA et al. GTDB-Tk: A toolkit to classify genomes with the genome taxonomy database. *Bioinformatics* 2020;**36**:1925–1927. https://doi.org/10.1093/bioinformatics/btz848

11. Oksanen J et al. vegan: Community Ecology. R package version 2.5-7. 2020.

12. Fernandes et al. Unifying the analysis of high-throughput sequencing datasets: characterizing RNA-seq, 16S rRNA gene sequencing and selective growth experiments by compositional data analysis. *Microbiome* 2014;**2**:1–13.

13. Gloor GB, Macklaim JM, Fernandes AD. Displaying Variation in Large Datasets: Plotting a Visual Summary of Effect Sizes. *J Comput Graph Stat* 2016;**25**:971–979. https://doi.org/10.1080/10618600.2015.1131161

Supplementary Tables

Tab. S1. Chemical analyses of porewater and solid sediments at Fehmarn Belt (stations 4, 11, 33) and Odra Bank (stations 44, 53, 62) (TC, total carbon; TIC, total inorganic carbon; TN, total nitrogen; TS, total sulfur; SRR, sulfate reduction rate; STD, standard deviation).

Station	Depth	Fe [mmol L^{-1}]	Mn [mmol L^{-1}]	H_2S [mmol L^{-1}]	SO_4 [mmol L^{-1}]	TC [% dry weight]	TIC [% dry weight]	TN [% dry weight]	TS [% dry weight]	SRR [nmol cm^{-3} d^{-1}]	STD SRR [nmol cm^{-3} d^{-1}]
4	0.5	0.09	0.18	0.00	16.06	3.25	0.18	0.36	0.41	4.68	3.40
4	1.5	0.25	0.29	0.00	15.90	2.77	0.09	0.32	0.42	20.41	7.52
4	2.5	0.62	0.27	0.00	16.37	NA	NA	NA	NA	32.80	32.02
4	3.5	0.76	0.36	0.00	15.99	2.25	0.11	0.24	0.40	22.10	5.74
4	4.5	0.36	0.23	0.00	16.05	2.55	0.16	0.28	0.50	27.43	17.33
4	5.5	0.44	0.31	0.00	16.01	NA	NA	NA	NA	31.93	17.63
4	6.5	0.13	0.19	0.00	16.24	2.08	0.09	0.22	0.61	NA	NA
4	7.00	NA	NA	NA	NA	NA	NA	NA	NA	27.65	26.71
4	8.5	0.01	0.14	0.01	16.00	NA	NA	NA	NA	NA	NA
4	9.0	NA	NA	NA	NA	NA	NA	NA	NA	13.20	8.69
4	10.5	0.00	0.11	0.05	16.77	NA	NA	NA	NA	NA	NA
4	12.5	0.00	0.08	0.10	16.59	NA	NA	NA	NA	12.23	9.87
4	14.5	0.00	0.07	0.14	16.97	NA	NA	NA	NA	NA	NA
4	17.5	NA	NA	NA	NA	NA	NA	NA	NA	1.80	1.03
4	19.5	0.00	0.06	0.25	17.52	NA	NA	NA	NA	NA	NA
4	25.5	0.00	0.04	0.36	17.28	NA	NA	NA	NA	NA	NA
4	30.5	0.00	0.04	0.45	16.82	NA	NA	NA	NA	NA	NA
11	0.5	0.21	0.16	0.00	16.15	3.59	0.30	0.41	0.52	5.67	2.81
11	1.5	0.14	0.15	0.00	16.00	3.73	0.18	0.45	0.67	10.89	3.90
11	2.5	0.17	0.34	0.00	15.77	NA	NA	NA	NA	12.99	2.79
11	3.5	NA	NA	NA	NA	2.92	0.11	0.32	0.86	10.82	4.27
11	4.5	0.06	0.28	0.01	15.32	2.78	0.10	0.30	0.72	12.10	5.20
11	5.5	0.02	0.19	0.01	16.13	NA	NA	NA	NA	10.93	7.42
11	6.5	0.01	0.22	0.02	16.21	4.57	0.30	0.52	1.54	NA	NA
11	7	NA	NA	NA	NA	NA	NA	NA	NA	9.33	4.71
11	8.5	0.00	0.16	0.09	16.12	NA	NA	NA	NA	NA	NA
11	9	NA	NA	NA	NA	NA	NA	NA	NA	6.77	3.14
11	10.5	0.00	0.13	0.17	15.97	NA	NA	NA	NA	NA	NA
11	12.5	0.00	0.12	0.10	16.10	NA	NA	NA	NA	4.44	1.85
11	14.5	0.00	0.10	0.24	16.02	NA	NA	NA	NA	NA	NA
11	17.5	NA	NA	NA	NA	NA	NA	NA	NA	3.66	2.33
11	20.5	0.00	0.07	0.55	15.49	NA	NA	NA	NA	NA	NA
11	25.5	0.00	0.06	0.65	15.56	NA	NA	NA	NA	NA	NA
33	0.5	0.00	0.16	0.00	16.55	3.01	0.16	0.35	0.37	1.74	0.64
33	1.5	0.01	0.50	0.00	16.68	3.25	0.16	0.38	0.41	18.49	16.72
33	2.5	0.27	0.77	0.00	16.48	NA	NA	NA	NA	19.46	12.33
33	3.5	0.33	0.79	0.00	16.35	5.07	0.22	0.62	0.63	20.09	10.04
33	4.5	0.35	0.67	0.00	16.12	3.61	0.14	0.43	0.48	25.67	10.34
33	5.5	0.36	0.68	0.00	16.01	NA	NA	NA	NA	28.10	11.38
33	6.5	NA	NA	NA	NA	2.88	0.15	0.33	0.52	NA	NA
33	7.0	NA	NA	NA	NA	NA	NA	NA	NA	61.54	37.50
33	7.5	0.05	0.49	0.00	15.34	NA	NA	NA	NA	NA	NA
33	9.0	NA	NA	NA	NA	NA	NA	NA	NA	63.15	34.56
33	9.5	0.00	0.29	0.04	16.03	NA	NA	NA	NA	NA	NA
33	11.5	0.00	0.15	0.12	15.92	NA	NA	NA	NA	NA	NA
33	12.5	NA	NA	NA	NA	NA	NA	NA	NA	9.77	7.14
33	13.5	0.00	0.12	0.26	15.57	NA	NA	NA	NA	NA	NA
33	15.5	0.00	0.09	0.27	14.82	NA	NA	NA	NA	NA	NA
33	17.5	NA	NA	NA	NA	NA	NA	NA	NA	1.85	1.64
33	21.5	0.00	0.07	0.57	15.00	NA	NA	NA	NA	NA	NA
33	27.5	0.00	0.06	0.65	14.71	NA	NA	NA	NA	NA	NA
44	0.5	0.00	0.00	0.00	6.56	0.08	0.00	0.01	0.01	0.11	0.19
44	1.5	0.00	0.00	0.00	6.61	0.14	0.01	0.01	0.01	0.85	1.47
44	2.5	0.00	0.00	0.00	6.72	NA	NA	NA	NA	1.81	1.60
44	3.5	0.00	0.00	0.00	6.71	0.19	0.00	0.01	0.02	13.38	21.17
44	4.5	0.00	0.00	0.00	6.83	0.19	0.01	0.01	0.01	26.55	44.58
44	5.5	NA	NA	NA	NA	NA	NA	NA	NA	15.96	26.55
44	6.5	0.00	0.00	0.00	6.65	0.34	0.21	0.01	0.02	NA	NA
44	7.0	NA	NA	NA	NA	NA	NA	NA	NA	14.96	19.92

44	8.5	0.00	0.00	0.00	6.77	NA	NA	NA	NA	NA	NA
44	9.0	NA	NA	NA	NA	NA	NA	NA	NA	23.82	20.33
44	10.5	0.00	0.00	0.00	6.86	NA	NA	NA	NA	NA	NA
44	12.5	0.00	0.00	0.00	6.85	NA	NA	NA	NA	22.08	6.93
44	14.5	0.00	0.00	0.00	6.76	NA	NA	NA	NA	NA	NA
44	15.5	NA	NA	NA	NA	NA	NA	NA	NA	NA	NA
44	16.5	0.00	0.00	0.00	6.75	NA	NA	NA	NA	NA	NA
44	17.5	NA	NA	NA	NA	NA	NA	NA	NA	13.18	6.98
44	18.5	0.00	0.00	0.00	6.51	NA	NA	NA	NA	NA	NA
44	20.5	0.00	0.00	0.00	6.36	NA	NA	NA	NA	NA	NA
53	0.5	0.00	0.00	0.00	7.07	0.13	0.00	0.01	0.01	0.20	0.35
53	1.5	0.00	0.00	0.00	7.02	0.09	0.00	0.01	0.01	1.22	2.03
53	2.5	0.00	0.00	0.00	6.87	NA	NA	NA	NA	1.50	2.43
53	3.5	0.00	0.00	0.00	6.85	0.28	0.02	0.02	0.01	0.64	0.96
53	4.5	0.01	0.00	0.00	6.24	0.19	0.00	0.02	0.02	13.17	11.29
53	5.5	NA	NA	NA	NA	NA	NA	NA	NA	20.91	12.83
53	6.5	0.02	0.00	0.00	6.84	0.15	0.05	0.01	0.00	NA	NA
53	7.0	NA	NA	NA	NA	NA	NA	NA	NA	35.69	26.93
53	8.5	0.01	0.00	0.00	6.97	NA	NA	NA	NA	NA	NA
53	9.0	NA	NA	NA	NA	NA	NA	NA	NA	26.01	19.27
53	10.5	0.01	0.00	0.00	7.06	NA	NA	NA	NA	NA	NA
53	12.5	0.01	0.00	0.00	7.20	NA	NA	NA	NA	6.72	1.81
53	14.5	0.01	0.00	0.00	7.35	NA	NA	NA	NA	NA	NA
53	16.5	0.01	0.00	0.00	7.18	NA	NA	NA	NA	NA	NA
53	17.5	NA	NA	NA	NA	NA	NA	NA	NA	7.83	6.44
53	18.5	0.02	0.00	0.00	7.16	NA	NA	NA	NA	NA	NA
62	0.5	0.00	0.00	0.00	6.56	0.11	0.00	0.01	0.01	0.00	0.00
62	1.5	0.00	0.00	0.00	6.14	0.18	0.06	0.01	0.00	0.02	0.04
62	2.5	0.00	0.00	0.00	6.13	NA	NA	NA	NA	0.23	0.13
62	3.5	0.00	0.00	0.00	6.25	0.14	0.00	0.01	0.01	1.52	0.77
62	4.5	0.00	0.00	0.00	6.40	0.24	0.00	0.01	0.02	3.60	2.38
62	5.5	NA	NA	NA	NA	NA	NA	NA	NA	2.74	1.14
62	6.5	0.00	0.00	0.00	6.14	0.09	0.00	0.01	0.01	NA	NA
62	7.0	NA	NA	NA	NA	NA	NA	NA	NA	9.57	11.07
62	8.5	0.00	0.00	0.00	6.41	NA	NA	NA	NA	NA	NA
62	9.0	NA	NA	NA	NA	NA	NA	NA	NA	7.00	4.47
62	10.5	0.00	0.00	0.00	6.49	NA	NA	NA	NA	NA	NA
62	12.5	0.00	0.00	0.00	6.33	NA	NA	NA	NA	4.61	0.68
62	14.5	0.00	0.00	0.00	6.37	NA	NA	NA	NA	NA	NA
62	16.5	0.00	0.00	0.00	6.49	NA	NA	NA	NA	NA	NA
62	17.5	NA	NA	NA	NA	NA	NA	NA	NA	1.01	0.60
62	18.5	0.00	0.00	0.01	6.71	NA	NA	NA	NA	NA	NA
62	20.5	0.00	0.00	0.01	6.86	NA	NA	NA	NA	NA	NA

Tab S2. Percentages of *dsrA* transcripts in metatranscriptomes of Fehmarn Belt (FB) and Odra Bank (ODB) assigned to prokaryotic orders using the GTDB taxonomy (r232) (min, minimum percentage; max, maximum percentage; mean, average percentage across all samples of the respective area).

Phylum	Class	Order	min FB	max FB	mean FB	min ODB	max ODB	mean ODB	mean all
Desulfobacterota	Desulfobacteria	Desulfobacterales	35.56	61.53	49.90	24.49	59.10	42.73	46.76
uncl_Bacteria	uncl_Bacteria	uncl_Bacteria	14.62	22.08	18.31	0.00	12.54	7.73	13.68
Nitrospirota	Thermodesulfovibrionia	Thermodesulfovibrionales	0.27	4.47	1.51	8.77	60.30	27.94	13.07
Desulfobacterota	Desulfobulbia	Desulfobulbales	0.25	29.31	10.10	0.00	9.05	4.27	7.55
Nitrospirota	uncl_Nitrospirota	uncl_Nitrospirota	0.00	0.00	0.00	1.15	10.80	7.08	3.10
Desulfobacterota	uncl_Desulfobacterota	uncl_Desulfobacterota	1.09	3.85	2.62	0.00	3.86	2.15	2.41
Desulfobacterota	Syntrophobacteria	BM002	0.43	10.28	2.81	0.00	4.58	1.55	2.26
Desulfobacterota	DSM-4660	Desulfatiglandales	1.33	4.71	2.93	0.00	1.68	0.50	1.86
Myxococcota_A	Polyangia	JAAYKL01	0.07	2.84	1.58	0.00	1.17	0.30	1.02
Desulfobacterota	Desulfobaccia	Desulfobaccales	0.00	0.52	0.06	0.00	7.07	1.46	0.67
Nitrospirota	Thermodesulfovibrionia	uncl_Thermodesulfovibrionia	0.00	0.00	0.00	0.00	4.02	1.49	0.65
Gemmatimonadota	Gemmatimonadetes	Gemmatimonadales	0.00	1.08	0.60	0.00	2.73	0.64	0.62
Fibrobacterota	Fibrobacteria	UBA11236	0.00	1.73	0.39	0.00	1.17	0.59	0.48
RBG-13-61-14	RBG-13-61-14	RBG-13-61-14	0.00	1.75	0.81	0.00	0.13	0.02	0.46
Acidobacteriota	Thermoanaerobaculia	Thermoanaerobaculales	0.00	1.76	0.32	0.00	1.64	0.49	0.40
Planctomycetota	JAJSAM01	JAJSAM01	0.09	2.33	0.68	0.00	0.21	0.03	0.39
Chloroflexota	Dehalococcoidia	E44-bin15	0.00	2.24	0.65	0.00	0.11	0.03	0.38
Zixibacteria	MSB-5A5	GN15	0.00	1.41	0.59	0.00	0.37	0.05	0.36
Chloroflexota	Dehalococcoidia	Dehalococcoidales	0.00	1.50	0.59	0.00	0.28	0.04	0.35
Myxococcota_A	Polyangia	Polyangiales	0.00	1.68	0.46	0.00	0.30	0.04	0.28
Planctomycetota	uncl_Planctomycetota	uncl_Planctomycetota	0.00	0.97	0.48	0.00	0.06	0.01	0.27
Myxococcota	B64-G9	B64-G9	0.00	1.40	0.47	0.00	0.00	0.00	0.27
Desulfobacterota	Desulfarculia	Adiutricales	0.00	1.77	0.45	0.00	0.00	0.00	0.25
Planctomycetota	Planctomycetia	Pirellulales	0.00	0.39	0.16	0.00	1.45	0.35	0.25
Desulfobacterota	DSWW01	DSWW01	0.00	0.84	0.24	0.00	0.64	0.19	0.22
Krumholzibacteriota	Krumholzibacteriia	WVZY01	0.11	0.92	0.37	0.00	0.00	0.00	0.21
SAR324	SAR324	XYD2-FULL-50-16	0.00	0.97	0.36	0.00	0.00	0.00	0.20
Planctomycetota	PLA2	PLA2	0.00	1.18	0.30	0.00	0.30	0.06	0.20
Myxococcota_A	UBA9160	UBA9160	0.00	1.13	0.29	0.00	0.13	0.02	0.17
Chloroflexota	Dehalococcoidia	uncl_Dehalococcoidia	0.00	0.75	0.27	0.00	0.12	0.02	0.16
Bacteroidota	Ignavibacteria	Ignavibacteriales	0.00	1.10	0.26	0.00	0.00	0.00	0.15
JAUVQV01	JAUVQV01	JAUVQV01	0.00	0.95	0.18	0.00	0.00	0.00	0.10
Myxococcota_A	Polyangia	DRWM01	0.00	0.42	0.13	0.00	0.18	0.03	0.08
Abyssobacteria	SURF-5	SURF-5	0.00	0.41	0.13	0.00	0.00	0.00	0.07
UBA10199	UBA10199	UBA10199	0.00	0.36	0.11	0.00	0.00	0.00	0.06
Myxococcota_A	UBA796	UBA9615	0.00	0.35	0.10	0.00	0.13	0.02	0.06
Desulfobacterota	Desulfarculia	Desulfarculales	0.00	0.43	0.10	0.00	0.00	0.00	0.06
Desulfobacterota	Desulfarculia	uncl_Desulfarculia	0.00	0.80	0.09	0.00	0.00	0.00	0.05
Bdellovibrionota_B	Oligoflexia	CAYFLR01	0.00	0.34	0.08	0.00	0.00	0.00	0.05
Acidobacteriota	uncl_Acidobacteriota	uncl_Acidobacteriota	0.00	0.74	0.08	0.00	0.00	0.00	0.05
ARS69	ARS69	ARS69	0.00	0.68	0.08	0.00	0.00	0.00	0.05
Electryoneota	AABM5-125-24	Hatepunaeales	0.00	0.43	0.08	0.00	0.00	0.00	0.04
Planctomycetota	B15-G4	B15-G4	0.00	0.00	0.00	0.00	0.69	0.10	0.04
Acidobacteriota	Terriglobia	Bryobacterales	0.00	0.11	0.01	0.00	0.50	0.07	0.04
Chloroflexota	Dehalococcoidia	SZUA-161	0.00	0.27	0.06	0.00	0.00	0.00	0.04
Bacteroidota	Ignavibacteria	uncl_Ignavibacteria	0.00	0.30	0.06	0.00	0.00	0.00	0.03
Myxococcota	XYA12-FULL-58-9	XYA12-FULL-58-9	0.00	0.22	0.06	0.00	0.00	0.00	0.03
Electryoneota	Electryoneia	Electryoneales	0.00	0.24	0.05	0.00	0.00	0.00	0.03
Zixibacteria	MSB-5A5	uncl_MSB-5A5	0.00	0.20	0.03	0.00	0.00	0.00	0.02

Tab. S3. Additional taxonomic analysis of *dsrA* transcripts that remained unclassified at phylum level. Sequences were further examined by phylogenetic placement (epa-ng) (see Supplementary Methods section) and similarity searches through BLASTn using the sequence collection by Diao et al. (2023) and NCBI nt database, respectively (max_FB, max_ODB, mean_FB, mean_ODB: maximum and mean TPM of the respective transcript in Fehmarnbelt and Odra Bank metatranscriptomes, respectively; epa_taxpath: classification by epa-ng; DiaoDB_ID, DiaoDB_pident, DiaoDB_evalue, DiaoDB_bitscore, DiaoDB_taxpath: identifier of the best match, the corresponding e-value and bitscore, and the related GTDB classification obtained from similarity searches against the sequence collection by Diao et al. (2023), respectively; NCBI_ID, NCBI_pident, NCBI_evalue; NCBI_bitscore, NCBI_taxID, NCBI_taxpath: identifier of the best match, the corresponding e-value and bitscore, the NCBI taxID and the related NCBI classification obtained from similarity searches against the NCBI nt database.

See file TabS3.xlsx

Tab. S4. Summary of all transcribed genes with functional annotations and their average transcription levels. Gene abbreviations (as used in Figs. 8 and S5) are provided together with their respective KEGG Orthology (KO) identifiers. Average (avg) transcription levels for each site and depth interval are reported as transcripts per million (TPM) (FB, Fehmarn Belt; ODB, Odra Bank).

See file TabS4_revised.xlsx

Tab. S5. Gene content and transcriptional activity of *Thermodesulfovibrionia* MAG EMB267_AVHV_4_191. Expression levels for each gene in each sample are reported as transcripts per million (TPM), scaled to the total number of transcripts mapped to the MAG. For genes with KEGG annotations, the corresponding KEGG Orthology (KO) identifiers and functional descriptions are provided.

See file TabS5_revised.xlsx

Supplementary Figures

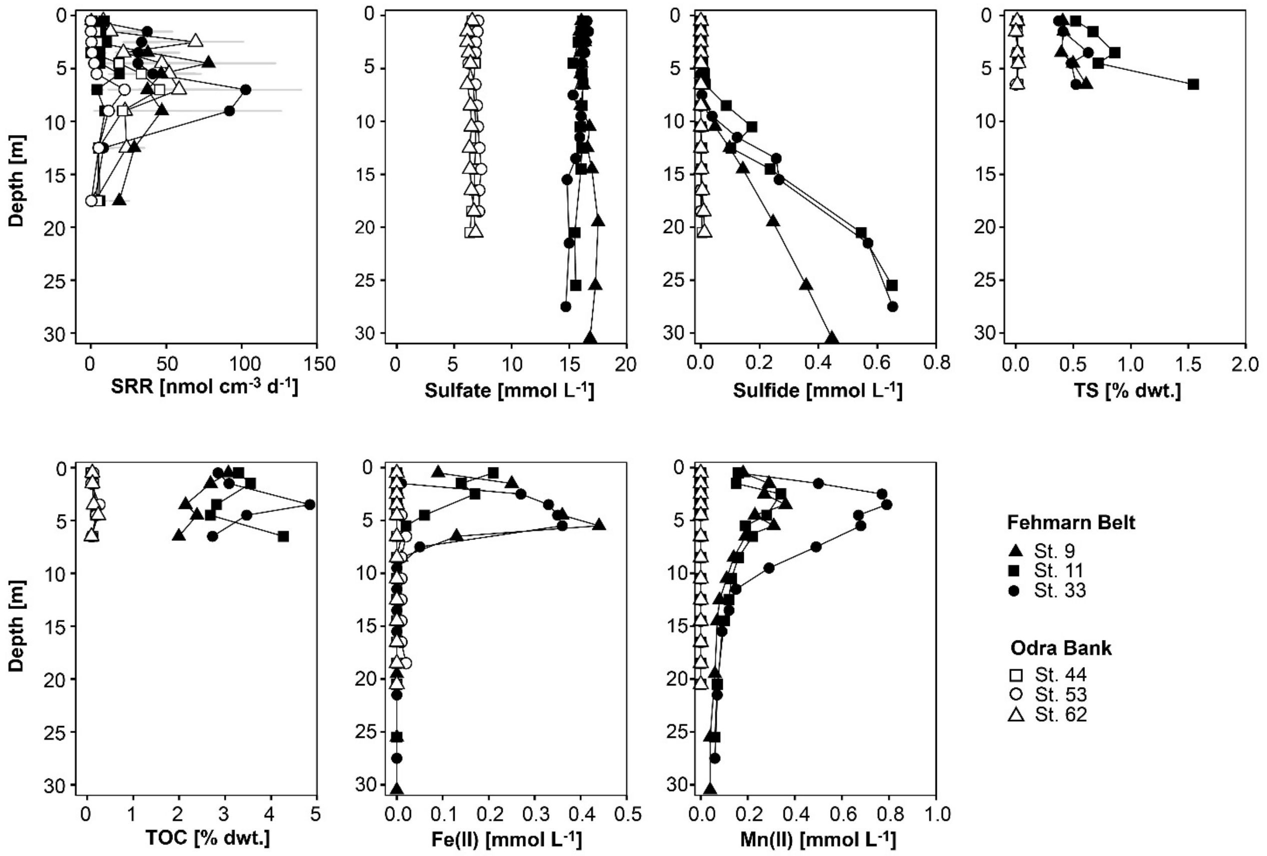

Fig. S1. Comparison of sulfate reduction rates (SRR), porewater constituents (Fe(II): dissolved iron; Mn(II): dissolved manganese), total sulfur (TS) and total organic carbon at the Fehmarn Belt and Odra Bank stations. Identical x-axis ranges and tick intervals were used for both locations to facilitate direct comparison.

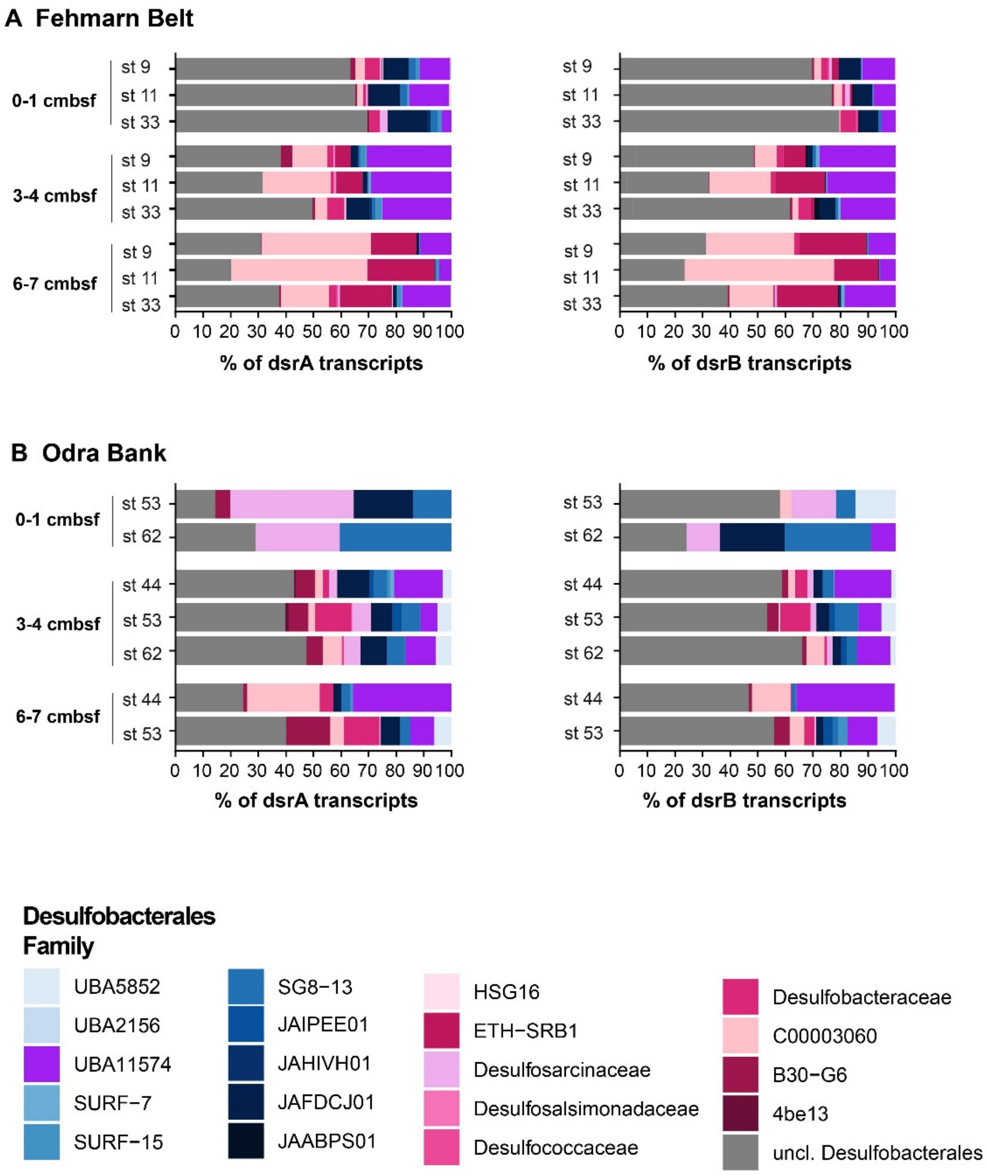

Fig. S2. Family-level classification of *Desulfobacterales dsrA* (left panels) and *dsrB* transcripts (right panels) in sediments from Fehmarn Belt (A) and Odra Bank (B) at depths of 0-1 centimeters below seafloor (cmbsf), 3-4 cmbsf and 6-7 cmbsf.

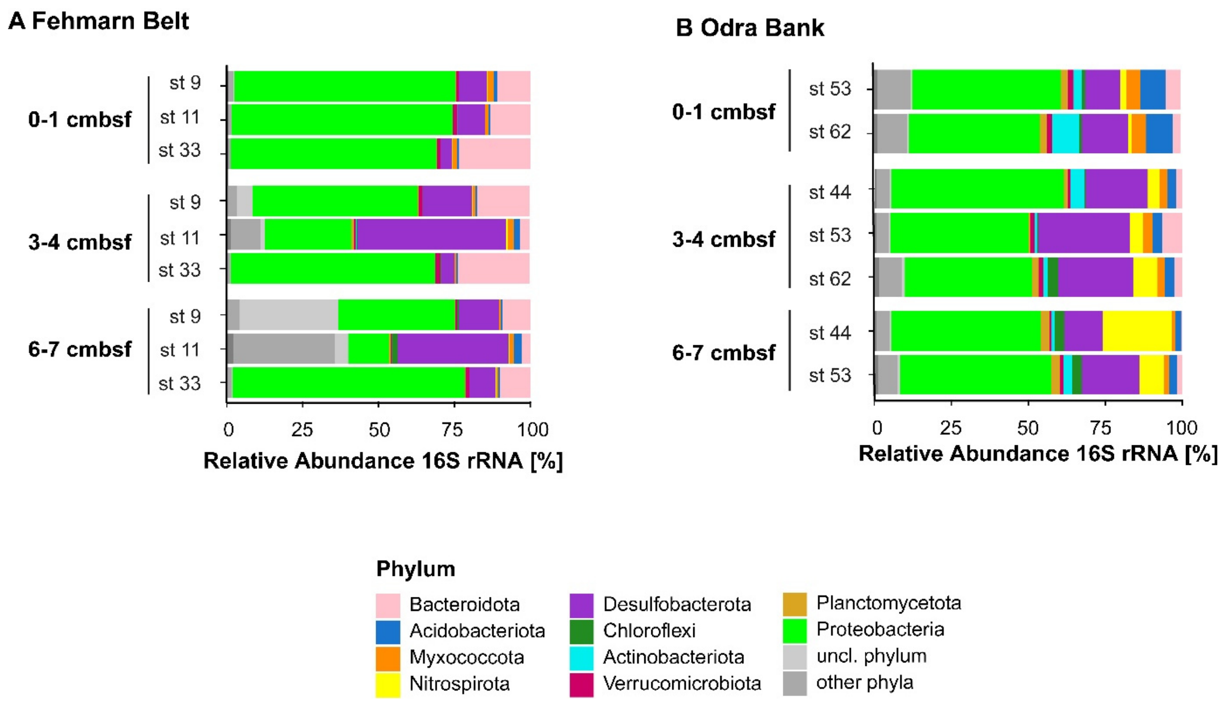

Fig. S3. Relative abundance of phyla based on 16S rRNA sequences in sediment samples from Fehmarn Belt (A) and Odra Bank (B). The ten most abundant phyla are represented by different colors. Phyla with lower abundance were summarized in the category “other phyla”.

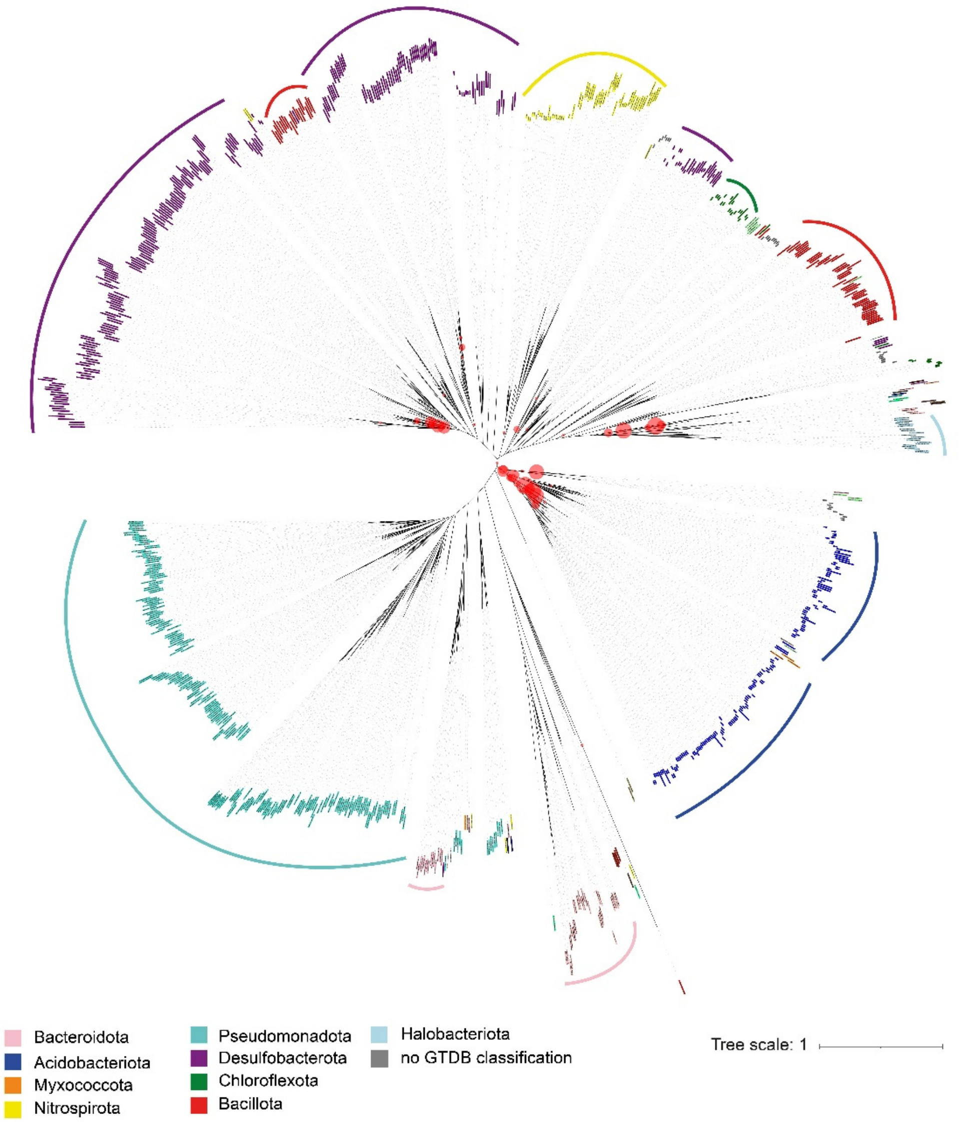

Fig. S4. Phylogenetic placement of dsrA sequences from our study that could not be classified at phylum level based on sequence similarity (indicated by the red circles). The tree was constructed from a published *dsrA* sequence compilation [3]

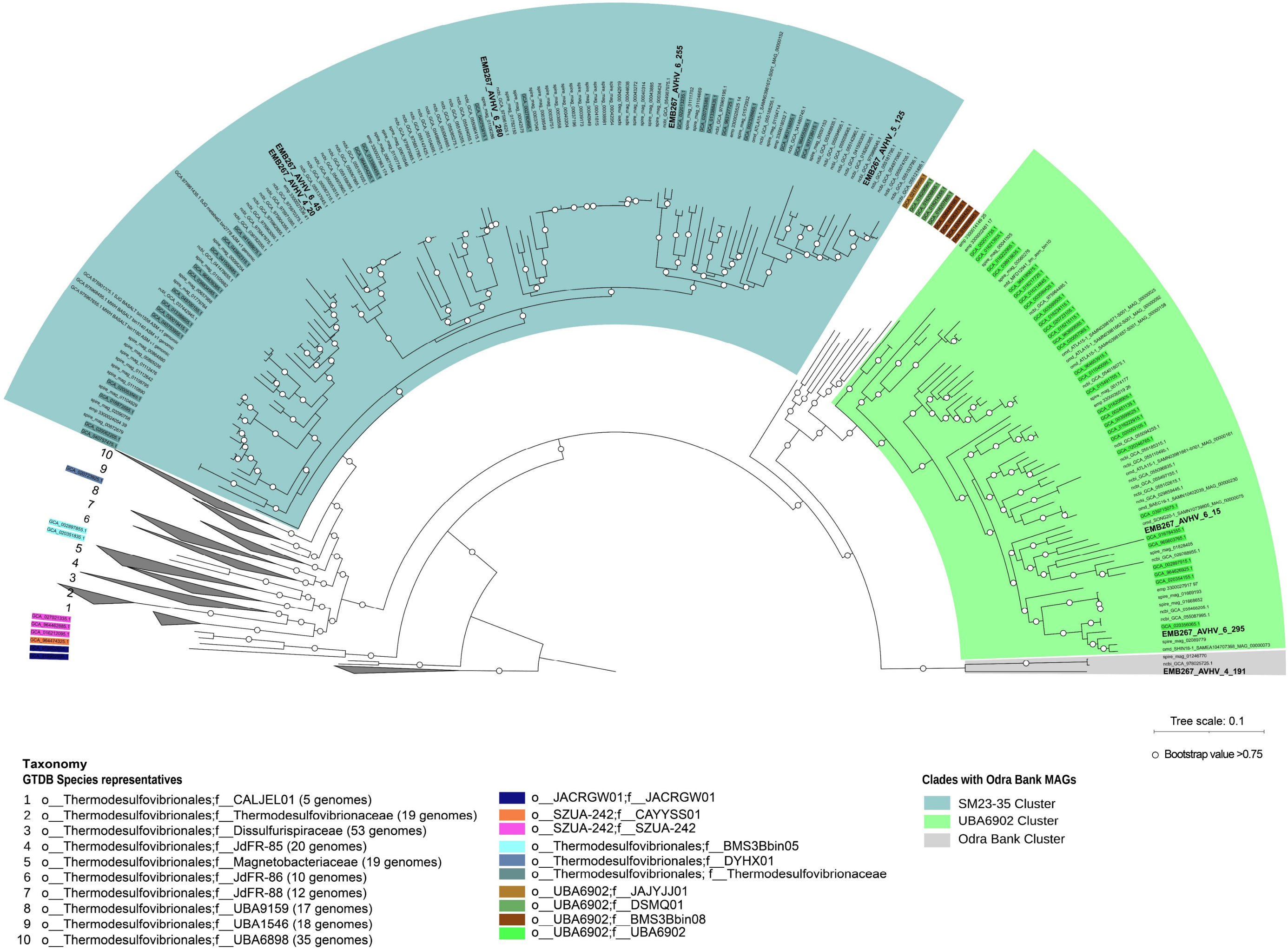

Fig. S5. Phylogenomic marker gene tree of *Thermodesulfovibrionia* including MAGS >60% completeness and <3% contamination from Odra Bank sediments, GTDB and published gene catalogues.

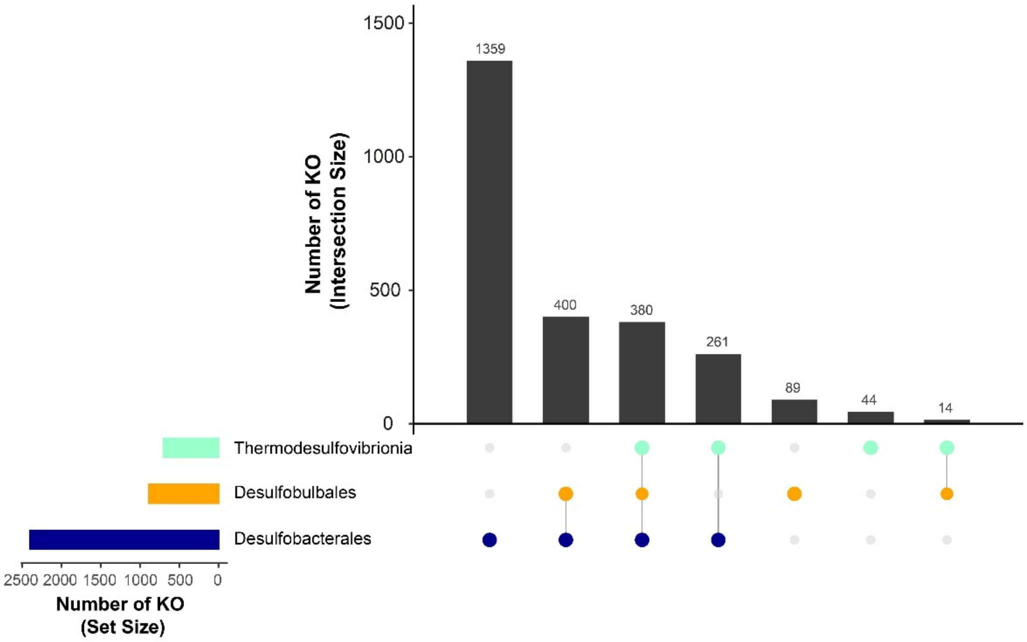

Fig. S6. Number of different KEGG orthologs assigned to the major *dsrAB*-transcribing lineages in Fehmarn Belt and Odra Bank samples.

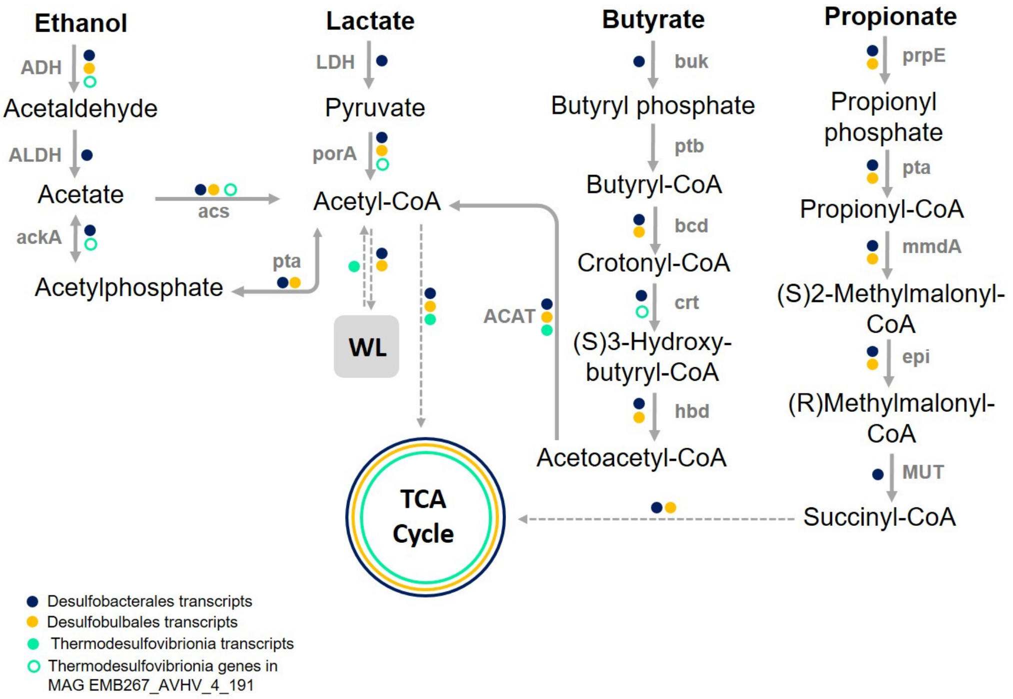

Fig. S7. Pathways of carbon metabolism encoded by transcripts of major sulfate-reducing lineages (WL, Wood-Ljungdahl pathway). Colours assigned to taxa according to Fig. 8. For abbreviations of gene names see Tab. S4.

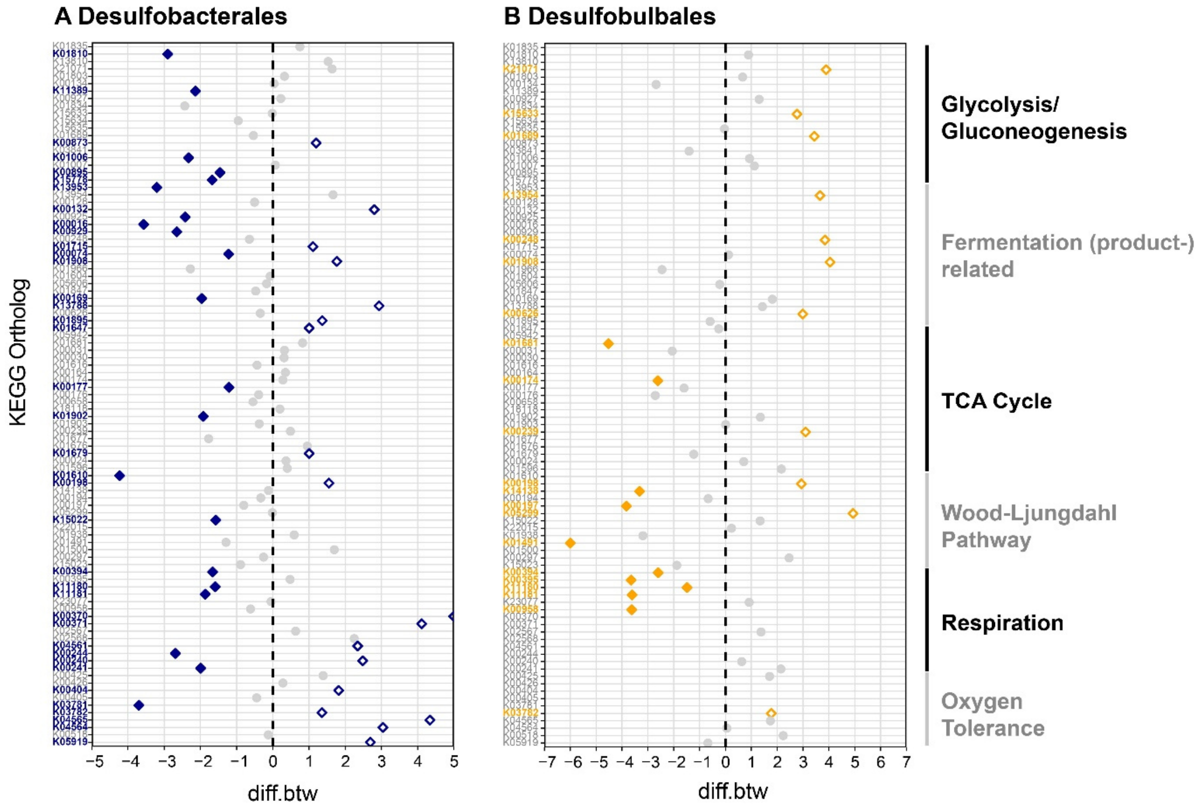

Fig. S8. Changes in the composition of functional gene transcripts between Fehmarn Belt and Odra Bank samples for (A) *Desulfobacterales* and (B) *Desulfobulbales*. Differentially abundant KEGG orthologs are highlighted in blue and orange for *Desulfobacterales* and *Desulfobulbales*, respectively. Negative and positive values for diff.btw indicate higher transcription levels at FB and ODB, respectively (see Supplementary Methods for further information).
