# Supplementary material for: A novel Thermodesulfovibrionia lineage largely supports sulfate reduction in sandy sediments of the southern Baltic Sea
Source: ISME Commun. 2026 Sep 4;6(1):ycag255. doi: 10.1093/ismeco/ycag255 (PMC13657043; doi:10.1093/ismeco/ycag255)
Supplement: Supplementary file 1 [file supplementary_material_ycag255.zip › MGF_SR_manuscript_for-proof2.docx]

**A novel *Thermodesulfovibrionia* lineage largely supports sulfate reduction in sandy sediments of the southern Baltic Sea**

Judith Piontek^1*^, Thomas Schott^1^, Patricia Roeser^1,2^, Jens Kallmeyer^3^, Michael Ernst Böttcher^1,4^, Christiane Hassenrück^1^, Klaus Jürgens^1^

^1^ Leibniz Institute for Baltic Sea Research Warnemünde, Seestr. 15, 18119 Rostock, Germany

^2^ Environmental Geology, Institute for Geosciences, University of Bonn, Kirschallee 1-3, 53115 Bonn, Germany

^3^ GFZ Helmholtz Centre for Geosciences, Telegrafenberg, 14473 Potsdam, Germany

^4^ Marine Geochemistry, University of Greifswald, Friedrich-Ludwig-Jahnstr., 17489 Greifswald, Germany

*Corresponding author: Judith Piontek ([judith.piontek@io-warnemuende.de](mailto:judith.piontek@io-warnemuende.de)), Leibniz Institute for Baltic Sea Research Warnemünde, Department of Biological Oceanography, Seestr. 15, 18119 Rostock, Germany

*Short title: Sulfate reduction in sandy sediments*

**Abstract**

Sandy sediments of the coastal ocean are generally not considered a habitat for highly active sulfate-reducing microorganisms due to fluctuations in oxygen concentrations and carbon availability. This study investigated the taxonomic composition and gene expression patterns of sulfate-reducing communities in both sandy, organic-poor sediments and muddy, organic-rich sediments in the mesohaline southern Baltic Sea. Our results revealed that a novel order-level lineage within the class *Thermodesulfovibrionia* (phylum *Nitrospirota*), named *Arenidesulfomonadales*, contributed significantly to sulfate reduction in the sandy sediments and supported sulfate reduction rates similar to those in the organic-rich muddy sediments. In the sands, *Thermodesulfovibrionia* effectively competed with *Desulfobacterales*, which strongly dominated the sulfate-reducing communities in the organic-rich sediments. Metatranscriptomic data revealed that *Desulfobacterales* functioned as typical secondary fermenters in both sediment types and expressed nearly complete sets of genes for the catabolism of fermentation products such as lactate, propionate, and butyrate. In contrast, the gene expression profile of *Thermodesulfovibrionia* suggests that hydrogenotrophic sulfate reduction, coupled with carbon fixation via the Wood-Ljungdahl pathway and a reduced capacity to oxidize organic compounds, was a competitive metabolic strategy enabling sulfate reduction to proceed independently of fermentation-derived carbon. Overall, our results suggest that overlooked lineages of sulfate-reducing microorganisms may contribute to higher levels of sulfate reduction than previously assumed in the extensive areas of permeable sediments of coastal and shelf seas.

**Key words:** sulfate reduction, marine sediments, metatranscriptomes, Thermodesulfovibrionia, Baltic Sea

**Introduction**

Sulfate-reducing microorganisms (SRM) are a key functional group of bacteria and archaea widely distributed in aquatic and terrestrial environments under anoxic and hypoxic conditions [1–4]. Their presence across diverse habitats and biogeographic regions reveals a wide range of ecophysiological tolerance for salinity, pH, and temperature that is founded on a broad phylogenetic range.

Well-characterized SRM are currently assigned to the phyla *Desulfobacterota* (previously referred to as *Deltaproteobacteria*)*, Bacillota*, *Nitrospirota* and *Thermodesulfobiota* [5, 6]. Over the past decade, culture-independent methods have substantially expanded the known diversity of SRM. Analysis of 950 mostly metagenome-assembled genomes (MAGs) suggested that SRM are found in 19 out of 23 bacterial and 2 out of 4 archaeal phyla [7]. However, less is known about the active metabolism of SRM in natural habitats and the contributions of both newly identified SRM and well-characterized cultivated taxa to *in-situ* sulfate reduction rates. The linkages between sulfate reduction and the microbial metabolism of carbon, nitrogen and redox-sensitive metals in these phylogenetically diverse communities are still poorly understood, despite the potentially significant implications for biogeochemical element cycling in benthic ecosystems.

In marine sediments, sulfate reduction and, therefore, the activity of SRM play a major role in the remineralization of organic carbon. Particularly in coastal oceans, which account for only 7% of the global ocean, a major fraction of 83% of the sedimentary organic carbon is processed [8, 9]. In these sediments, sulfate reduction can be quantitatively as important as, or even exceed, aerobic respiration in the turnover of organic matter [10]. Several studies identified members of the phylum *Desulfobacterota* as the most abundant taxonomic group of SRM in marine sediments, including sites in the North Sea - Baltic Sea transition zone, as well as the mesohaline and the oligohaline regions of the Baltic Sea [11–16]. However, most studies have focused on organic-rich muddy sediments, while organic-poor sands, which cover about 50-80% of the continental shelves, have received less attention [17, 18]. Although sulfate reduction rates in permeable sediments are assumed to be lower, a few studies have reported rates in the same range as those at organic-rich muddy sites [19, 20]. The low amount of organic matter, fluctuating oxygen concentrations, and hydrodynamic disturbance in permeable sands pose significant challenges for SRM [21–23]. However, given the large spatial extent of sandy sediments in coastal oceans, SRM adapted to the prevailing conditions could make a significant and hitherto unaccounted contribution to sulfate reduction.

In this study, we investigated the phylogenetic diversity and active metabolism of sulfate-reducing communities in Baltic Sea sediments with contrasting physical and chemical properties. We compared sulfate-reducing communities in muddy sediments from the Fehmarn Belt (FB) with those in sandy sediments from Odra Bank (ODB). The two sites differ markedly in grain size distribution, sedimentary organic carbon content and salinity. Our main objectives were to (1) identify the dominant active SRM, (2) characterize their metabolic activity through transcriptional profiles, and (3) disentangle site-specific shifts in both composition and function of sulfate-reducing communities.

**Material and Methods**

**Study Area and Sampling**

Sediment samples were taken in the southern Baltic Sea between 23 and 31 March 2022 during the cruise AL570 with RV Alkor (Fig. 1 A). A multicorer (MUC) was used to collect muddy sediments at three stations at FB at a water depth of 23 m (stations 9, 11, 33). Sandy sediments with a median grain size of approximately 180 µm were sampled at three stations at ODB at a water depth of 14 m (stations 44, 53, 62). In the bottom water, the salinity was 21 psu at FB and 8 psu at ODB. The oxygen concentrations ranged between 340 and 420 µmol L^-1^ (high-accuracy sensor SBE 43, Seabird Electronics, USA) and temperatures between 4.6 and 5.7°C.

Remote sensing data indicate that sediment sampling was conducted in both areas during the decline of the spring bloom. Maximum annual chlorophyll-a concentrations were reached approximately two to three weeks before sediment samples were collected (Fig. 1B).

For amplicon sequencing and metatranscriptomes, the MUC cores were subsampled with a tube of 3 cm in diameter. First, a surface sediment sample (0-1 cm) of the MUC core was taken under oxic conditions a few centimeters away from the smaller tube before the sub-core was removed and transferred to a glove bag. In the glove bag, the small sediment core was immediately cut into 1 cm sections (down to approx. 9 cmbsf) under an argon atmosphere to prevent contact between the anoxic subsurface sediments and the air. Samples were flash-frozen in liquid nitrogen and stored at -80°C until further processing. All steps of the subsampling were completed within 20 minutes.

**Geochemical analyses of porewater and sediment**

Iron, manganese and sulfur in the porewater samples were analyzed by ICP-OES (Thermo iCAP 7400 Duo, Thermo Fischer Scientific) using matrix-matched external calibration. Precision and accuracy were checked with spiked SLEW-3 (NRCC) and were better than ±4.7 and ±7.6%, respectively [24]. Samples for the sum of dissolved sulfide species (H_2_S) were preserved immediately after sampling with zinc acetate solution and stored cool and dark until analysis via spectrophotometry (Specord 40 spectrophotometer, Analytik Jena) [25]. Sulfate concentrations were obtained from the sulfur measurements, upon substraction of sulfide concentrations.

Freeze-dried sediments were analyzed for total carbon (TC), total nitrogen (TN), and total sulfur (TS) content with a CHNS Elemental Analyzer (Euro Vector EuroEA 3,052) as described previously [24]. Total inorganic carbon (TIC) was determined with an Elemental Analyzer multi-EA (Analytik Jena) after reaction with phosphoric acid followed by infrared spectrophotometry quantification of CO_2_. The precision of the method was ±3%, ±10%, ±7.6%, and ±4.3% for TC, TN, TS, and TIC, respectively. The content of total organic carbon (TOC) was calculated from the difference of TC and TIC.

**Sulfate reduction rates**

Sulfate reduction rates (SRR) were quantified with ^35^SO_4_^2-^ as a radiotracer using the whole-core incubation technique [26]. A single MUC core was used at each sampling site and subsampled with three 40 cm-long acrylic tubes (24 mm inner diameter) in order to have triplicate samples. The radiotracer (10 µL, activity ca. 200 kBq) was injected in 1 cm-intervals from the sediment-water interface down to 20 cm below sea floor (cmbsf). After injection, the tubes were incubated for 24 hrs at approximate in-situ temperature (ca. 10 to 15°C).

The incubations were terminated by pushing the sediment out of the acrylic core tubes, slicing them into depth sections and transferring the sediment into 50 ml centrifuge tubes, filled with 10 ml of 20% (w/v) zinc acetate solution. After returning to the home lab, the biologically produced radioactive reduced sulfur species were extracted by cold chromium distillation (see Supplementary Methods for details) [27]. Two types of control samples were prepared: For time-zero blanks, sediment samples were injected with radiotracer and the incubation was immediately terminated. For tracer blanks, the pure tracer was injected into zinc acetate solution. Subsequently, both types of blanks were processed like regular samples, and their measured values were subtracted from those of the samples. The limit of detection was around 0.4 nmol cm^-3^ d^-1^, always lower than the measured SRR.

**Extraction of nucleic acids**

RNA and DNA were co-extracted from the same subsample of 2 grams of sediment using the RNeasy PowerSoil Total RNA Kit combined with the DNA Elution Kit (both Qiagen, Hilden, Germany). All steps were performed according to the manufacturer's protocol, with the exception of the initial lysis of the samples, which was performed using a FastPrep bead beating grinder (MP Biomedicals) (twice 45 seconds at 6.5 m/s).

**Metatranscriptomics**

Metatranscriptomic libraries were generated from purified total RNA extracts after mixed rRNA depletion and sequenced on an Illumina NovaSeq 6000 (2x150 nt) at Genesupport SA (Plan-les-Ouates, Switzerland), yielding between 28 M and 36 M read pairs per sample. The paired end read libraries were adapter clipped and quality trimmed using bbduk (v39.01) [28]. Separately for the two sampling sites FB and ODB, the resulting read pairs were co-assembled using megahit (v1.3.3) (kmer list 37,47,57,67,77,87,97,107,117,127,137,147) (Li et al., 2015), before gene calling on contigs >200 nt was done using prodigal in meta-mode [29]. Predicted coding sequences were functionally annotated using diamond blastp against the Kyoto encyclopedia of genes and genomes (KEGG version 105) with a Blast-Score-Ratio cutoff of 0.4 [30–32]. Transcripts annotated as dissimilatory sulfite reductase (alpha subunit: K11180, beta subunit: K11181) were further characterized as reductive-type (involved in sulfate reduction) and reverse-type (involved in sulfide oxidation) using hmmer v3.4 (hmmer.org) against a curated set of sequences [33, 34]. Taxonomic classification was done using using CATpack (v6.0.1) (range parameter set to 3) against the genome taxonomy database (GTDB r232) [35, 36]. Reads per sample were mapped against the co-assembly using bowtie2 (v2.4.4) and then summarized using FeatureCounts of the Subread package (v 2.0.1) as transcripts per million (TPM) [37, 38].

**Amplicon Sequencing**

Partial 16S rRNA sequences were obtained from samples at 0-1 cmbsf, 3-4 cmbsf and 6-7 cmbsf at the six sampling stations at FB and ODB. Prior to sequencing, contaminating DNA in the RNA fraction was removed using the Turbo DNA-free kit (Thermo Fisher Scientific, Waltham, USA) and the 16S rRNA of each sample was transcribed into cDNA using the universal primer 1492R (5′-GGTTACCTTGTTACGACTT-3′) [39] and the MultiScribe Reverse Transcriptase (Thermo Fisher Scientific, Waltham, USA). The hypervariable V4–V5 region of the 16S rRNA sequence was amplified in the cDNA using the universal prokaryotic primers 515F-Y (5′-GTGYCAGCMGCCGCGGTAA) and 926R (5′-CCGYCAATTYMTTTRAGTTT) [40] and sequenced on an Illumina MiSeq platform at LGC Genomics GmbH (Berlin, Germany).

In addition to the 16S rRNA amplicon sequences, full-length 16S rRNA gene sequences were obtained from 6 samples (0-15 cmbsf) that were collected at the Odra Bank sampling site during a cruise in 2021 (EMB267 with RV Elisabeth Mann-Borgese). For this purpose, the 16S rRNA gene was amplified using the primer set 27F (5'-AGRGTTTGATYNTGGCTCAG-3') and 1492R (5'-TASGGHTACCTTGTTASGACTT-3') to generate HiFi reads on the PacBio Revio platform via circular consensus sequencing (Biomarker Technologies GmbH, Münster, Germany). These full-length 16SrRNA gene sequences were used exclusively to reinforce the phylogenetic analysis of *Thermodesulfivibrionia* (see the following section), while all compositional sequence analyses were derived from samples collected during the cruise AL570 as described above.

Both Illumina and PacBio reads were primer-clipped with cutadapt version 4.1 and quality-filtered and denoised in dada2 version 1.26.0 [41, 42]. In both cases, the taxonomic classification of the sequence variants was performed using the SILVA database (version 138.1) [43].

**Phylogenetic tree reconstruction for Thermodesulfovibrionia**

To explore phylogenetic relationships within the class *Thermodesulfovibrionia*, we compiled 150 non-redundant publicly available 16S rRNA gene sequences. The compilation contained environmental sequences archived in the SILVA collection, sequences recovered from genomes archived in GTDB and/or associated with publications [44, 45]. All sequences had a minimum length of 1000 nucleotides. For GTDB-derived MAGs, 16S rRNA gene sequences were retained only if the corresponding MAG exhibited <3% contamination. The compiled sequences were aligned together with 53 full-length 16S rRNA gene sequences obtained from the ODB samples using the SINA aligner (v1.2.12). Sequences showing poor alignment with the dataset were removed before a phylogenetic tree was constructed using FastTree with the GTR model of nucleotide evolution and Gamma20-based likelihood for modelling rate estimation as implemented in the Alignment, Classification and Tree Service provided by SILVA [46, 47]. The tree was rooted with seven 16S rRNA gene sequences affiliated with the class *Nitrospiria* and subsequently visualized and decorated in iToL [48].

Further analyses of sequence data, including phylogenetic placement and the statistical methods, are described in detail in Supplementary Methods.

**Results**

**Geochemical conditions and sulfate reduction in sediments of Fehmarn Belt and Odra Bank**

The sediments at FB and ODB differed markedly in their TOC, TN, and TS contents (Fig.2, Tab. S1, Fig. S1). At FB, the average TOC content was 3.1% dwt., which was about twenty times higher than the TOC concentrations at ODB (0.15% dwt.). Likewise, the corresponding TN contents at FB (0.37 % dwt.) were substantially higher than at ODB (≤0.01% dwt.). The accumulated sedimentary sulfur (essentially pyrite), a net product from microbial sulfate reduction, followed this trend (Fig. 2, Tab. S1, Fig. S1).

Several porewater constituents showed strongly different vertical profiles between the two sampling sites. Dissolved sulfide concentrations, as a first direct product of microbial dissimilatory sulfate reduction in the sediments, increased below 8 cmbsf to 0.36-0.65 mmol L^-1^ at 26-28 cmbsf at FB, whereas they remained ≤0.02 mmol L^-1^ from the surface down to 20 cmbsf at ODB (Fig. 2, Tab. S1, Fig. S1). In the porewaters, sulfate concentrations between 14.7 and 17.5 mmol L^-1^ at FB and between 6.1 and 7.2 mmol L^-1^ at ODB were rather constant over depth and mirrored the differences in salinity between the two stations. Dissolved iron and manganese showed maximum concentrations of up to 0.76 mmol Fe L^-1^ and 0.79 mmol Mn L^-1^ in the non-sulfidic top 8 cm of the FB sediments, while they remained below 0.02 mmol Fe L^-1^ and did not exceed 0.002 mmol Mn L^-1^ down to 20 cmbsf at ODB (Fig. 2, Tab. S1, Fig. S1).

Sulfate reduction rates (SRR) were variable between the stations but not significantly different between FB and ODB. Integrating the SRR over the top 20 cm of the sediment resulted in similar rates of 1.5-6.7 mmol m^-2^ d^-1^ at the two sites (Welch՚s t-test, t = 0.59, df = 3.98, p = 0.59). Maximum volumetric rates of 10.3-78.0 nmol cm^-3^ d^-1^ were determined at 2-8 cmbsf (Fig. 2, Fig. S1).

The proportions of transcripts encoding the alpha and beta subunit of the dissimilatory sulfite reductase (dsrAB), the marker enzyme of dissimilatory sulfate reduction, were significantly higher in FB sediments than at ODB (Welch՚s t-test, dsrA: t = 3.92, df = 13.97, p = 0.002, dsrB: t = 3.95, df = 13.93, p = 0.001) and exhibited less variability between stations compared to the sulfate reduction rates. Maximum dsrA proportions were found in the samples from 3-4 cmbsf or 7-8 cmbsf, the depth range in which the highest SRR were also measured. Consistent with the lowest observed SRR, the lowest proportions of *dsrA* transcripts were detected at 0–1 cmbsf in ODB sediments (Fig. 2).

**Taxonomic affiliation of dsrAB transcripts and proportions of major sulfate-reducing lineages in Fehmarn Belt and Odra Bank sediments**

Taxonomic assignments of transcripts at the order level revealed that overall at least 39 bacterial orders actively expressed genes encoding *dsrA* (Fig. 3, Tab. S2). Despite this broad diversity, the largest proportion of transcripts was assigned to a few dominant orders. At FB, *Desulfobacterales* and *Desulfobulbales* (both phylum *Desulfobacterota*) contributed together 44-68% to *dsrA* and 53-87% to *dsrB* transcripts (Fig. 3, Fig. S2). Proportions of *Desulfobacterales* dsrAB at ODB were comparable to those at FB, whereas *Desulfobulbales* dsrAB was reduced to 0-9% (Fig. 3, Tab. S2, Fig. S2). In contrast, ODB sediments were characterized by high proportions of *dsrAB* transcripts assigned to the class *Thermodesulfovibrionia* (phylum *Nitrospirota*) (Fig. 3). *Thermodesulfovibrionales* together with members of the *Thermodesulfovibrionia* unclassified at the order level contributed up to 60% to dsrA and 68% to dsrB at ODB, while the share of *Thermodesulfovibrionia* dsrAB was ≤4.5% in all FB samples (Tab. S2). These differences in *Thermodesulfovibrionia* dsrAB between FB and ODB coincided with corresponding differences in 16S rRNA sequence proportions assigned to the phylum *Nitrospirota* (Fig. S3).

A considerable fraction of *dsrA* and *dsrB* transcripts could not be taxonomically classified at the phylum level by similarity searches (FB: 14-22% of dsrA, ODB: 0-13% dsrA) (Fig. 3). Based on phylogenetic placement, at least a phylum level classification could be inferred for an average of 52% and 84% of these previously unclassified dsrA transcripts in FB and ODB samples, respectively, with sequences affiliated with *Desulfobacterota* predominating at both sites (Fig. S4, Tab. S3). This would increase the dsrA proportions originating from *Desulfobacterota* (shown in Fig. 3) by 1-20% and 0-10% in FB and ODB samples, respectively. Furthermore, dsrA proportions originating from *Myxococcota* and *Acidobacteriota* were slightly underestimated (Fig. S4, Tab. S3). However, even when accounting for the additional proportions inferred from phylogenetic placement, the dsrA proportions of *Myxococcota* and *Acidobacteriota* each contributed at most 8% to the *dsrA* transcripts in our samples.

Amplicon sequences of the 16S rRNA and the 16S rRNA gene provided further insights into the composition of the most important sulfate-reducing lineages (Figs. 4-6, Fig. S2). The 16S rRNA sequences showed a higher richness of *Desulfobacterales*, which comprised 1626 ASVs across all samples, than of *Desulfobulbales*, which included 237 ASVs. The composition of *Desulfobacterales* at the family and genus level was similar at FB and ODB and varied only slightly across sediment depths (Fig. 4). At both sampling sites, *Desulfobacterales* were strongly dominated by the genus Sva0081 sediment group (35-66%) and other genera of the family *Desulfosarcinaceae* in relatively constant proportions. In contrast, *Desulfobulbales* showed differences between the two sampling sites at family level. While *Desulfobulbaceae* (29–60%) were the dominant family of *Desulfobulbales* in most FB samples, ODB samples showed a higher proportion of *Desulfocapsaceae* (41–84%) (Fig. 4).

In contrast to *Desulfobacterales* and *Desulfobulbales*, none of the 51 ASVs of *Thermodesulfovibrionia* in the 16S rRNA sequences of the ODB samples could be classified at the order level using the SILVA database. The reconstruction of a phylogenetic tree from full-length 16S rRNA gene sequences of *Thermodesulfovibrionia* revealed that twenty-three 16S rRNA gene variants from Odra Bank sediments clustered with GTDB reference sequences of the family SM23-35 (order *Thermodesulfovibrionales*) together with unclassified environmental sequences mainly from sediments of freshwater and brackish systems (Fig. 5). Nine 16S rRNA gene variants from ODB samples formed a cluster with GTDB reference sequences of the family UBA6902 (order UBA6902) and environmental sequences mainly from marine sediments. Nineteen ODB 16S rRNA gene variants formed a well-supported monophyletic group together with ten environmental sequences from freshwater and fjord sediments of the Baltic Sea region and branched as a sister clade to the order UBA6902 (Fig. 5). This Odra Bank Cluster does not include any 16S rRNA sequence from GTDB genomes.

Notably, 85% of the *Thermodesulfovibrionia* 16S rRNA amplicon sequences were 100% identical to at least one of the full-length 16S rRNA gene sequences of the Odra Bank cluster, revealing that this cluster was the dominant *Thermodesulfovibrionia* lineage in the ODB sediments (Fig. 5-6). Substantially lower proportions of 3% and 0.5% of the 16S rRNA sequences matched with the clusters that represent the families SM23-35 (order *Thermdesulfovibrionales*) and UBA6902 (order UBA6902), respectively (Fig. 5-6).

A phylogenomic marker gene tree that includes seven high-quality *Thermodesulfovibrionia* MAGs from ODB sediments reflected the structure of the 16S rRNA gene tree and supports the existence of an Odra Bank Cluster (Fig. S5) [49]. MAG EMB267_AVHV_4_191, whose 16S rRNA gene sequence fell into the Odra Bank cluster of the 16S rRNA gene tree (Fig. 5), forms a monophyletic group in the phylogenomic marker gene tree together with two genomes from Mississippi River estuarine sediments that were obtained from public gene catalogues (Fig. S5). A value of 0.474 for the relative evolutionary divergence between this monophyletic group and the order UBA 6902 indicates that the Odra Bank Cluster represents an order-level lineage in the class *Thermodesulfovibrionia*. MAG EMB267_AVHV_4_191 recruited 71±28% of the metatranscriptomic reads from ODB subsurface samples that were assigned to *Thermodesulfovibrionia*, suggesting that it is representative of a large part of the active *Thermodesulfovibrionia* at this site (Fig. S5).

**Description of the Odra Bank Cluster as novel taxonomic lineage**

Based on the evidence obtained from the phylogenomic tree, we propose the name *Arenidesulfomonadales* ord. nov. for the ODB Cluster as the novel order within the class *Thermodesulfovibrionia*. MAG EMB267_AVHV_4_191 (GCA_985800395) was selected as nomenclatural type to define the type species *Arenidesulfomonas baltica* sp. nov. representing this new order ([A.re.ni.de.sul.fo.mo'nas] L. fem. n. arena, sand; N.L. pref. desulfo-, desulfuricating, here referring to sulfate reduction; L. fem. n. monas, a single unit; [bal.ti'ca] N.L. fem. adj. baltica , pertaining to the Baltic Sea or Baltic region). The name was chosen to indicate its origin from sandy sediments in the Baltic Sea and its role in this environment as key sulfate reducer based on functional gene annotation and gene expression analysis. Furthermore, we propose the genus *Arenidesulfomonas* gen. nov. and the family *Arenidesulfomonadaceae* fam. nov. to complete the lineage of the type species.

**Gene expression profiles of major sulfate-reducing taxa in Fehmarn Belt and Odra Bank sediments**

The active metabolism of the major *dsrAB*-transcribing lineages in FB and ODB sediments was inferred from the functional annotations of transcripts. Across all samples, 2400 different KEGG orthologs (KOs) were assigned to *Desulfobacterales*, whereas the other major sulfate-reducing taxa showed a lower functional diversity (*Desulfobulbales*: 883 KOs, *Thermodesulfovibrionia*: 699 KOs) (Fig. S6).

A two-dimensional NMDS plot based on Bray-Curtis dissimilarities resolved differences in the functional composition of transcripts between the major sulfate-reducing taxa and between FB and ODB samples (Fig. 7). PERMANOVA confirmed significant differences between the taxa and between the sampling sites (Taxonomic affiliation: *R^2^*=0.25, *F*_2,38_=6.35, p<0.001; Sampling site: *R^2^*=0.06, *F*_1,38_=3.15, *p*<0.001) (Fig. 7). It is noteworthy that high Bray-Curtis dissimilarities of up to 82% were found between transcripts of surface (0-1 cmbsf) and subsurface samples (3-4 cmbsf and 6-7cmbsf) across all sulfate-reducing lineages and at both sites (Fig. 7). *Desulfobacterales* and *Desulfobulbales* were present at both sampling sites, allowing a comparison of the functional composition of their transcripts at FB and ODB. *Desulfobulbales* showed stronger transcriptional differences between FB and ODB (mean Bray-Curtis dissimilarity 74±10%) than *Desulfobacterales* (46±14%).

Metatranscriptomes revealed differences in the expression of key genes for carbon and energy metabolism between the major sulfate-reducing lineages in our study. *Desulfobacterales* and *Desulfobulbales* expressed various genes that encode enzymes for the oxidation of primary fermentation products. In contrast, *Thermodesulfovibrionia* transcripts did not include any of the key enzymes for the oxidation of ethanol, lactate, butyrate and propionate (Fig. 8 A, Fig. S7, Tab. S4). Consistent with this finding, MAG EMB267_AVHV_4_191, representing *Arenidesulfomonadales* ord. nov. and recruiting the majority of *Thermodesulfovibrionia* transcripts, also contains only one gene encoding an alcohol dehydrogenase (Tab. S5, Fig. S7). Transcripts of acetyl-CoA C-acetyltransferase (ACAT) and enzymes involved in the first oxidation step of the TCA cycle suggests that acetoacetyl-CoA and acetyl-CoA were nevertheless relevant intermediates in the active *Thermodesulfovibrionia* metabolism in ODB sediments (Fig. 8 A, Fig. S7, Tab. S4).

All major sulfate-reducing lineages in our study expressed marker genes of the Wood-Ljungdahl pathway, which can operate bidirectionally either to completely oxidize acetate or to fix dissolved inorganic carbon, respectively (Fig. 8 A, Tab. S4).

The presence of marker genes of alternative respiratory processes implies that the major sulfate-reducing lineages used other electron acceptors in addition to sulfate (Fig. 8 B). *Desulfobacterales*, *Desulfobulbales* and *Thermodesulfovibrionia* transcribed genes encoding Frd, which can convert fumarate to succinate in anaerobic respiration. Furthermore, *Desulfobacterales* transcripts encoded marker genes of nitrate reduction (*narGH*, *napAB*, *norB*). Marker genes of both fumarate respiration and nitrate reduction were transcribed at higher levels in ODB than in FB sediments by *Desulfobacterales* (Fig. 8 B, Fig. S8, Tab. S4).

The major sulfate-reducing lineages showed substantially different proportions of hydrogenases in their transcriptomes. Low hydrogenase proportions of <0.01 to 0.02% of functionally annotated transcripts were found for *Desulfobacterales* and *Desulfobulbales*, whereas *Thermodesulfovibrionia* transcripts contained higher hydrogenase shares of 0.69% (Fig. 8 C). Most of the hydrogenase transcripts affiliated with *Desulfobacterales* (35%) and T*hermodesulfovibrionia* (47%) were classified as [NiFe]hydrogenase group 1a. The largest fraction of transcripts from *Desulfobulbales* (83%) were assigned to group 1c, which also represented a considerable fraction of the *Desulfobacterales* hydrogenase transcripts (30%) but accounted for less than 1% in the *Thermodesulfovibrionia* transcripts [50] (Fig. 8 C). In contrast, *Thermodesulfovibrionia* exhibited elevated proportions of [NiFe]hydrogenase group 1d transcripts, which constituted only a minor fraction in *Desulfobacterales* transcripts (1%) and were completely absent from the *Desulfobulbales* transcript pool (Fig. 8 C).

*Desulfobacterales*, *Desulfobulbales* and *Thermodesulfovibrionia* expressed several genes likely involved in the defence against reactive oxygen species (ROS) (Fig. 8 D, Tab. S4). All three taxa transcribed genes encoding catalase-peroxidase (katG), superoxide dismutase (SOD2), superoxide reductase (dfx), and cytochrome bd ubiquinol oxidase (cydA). The broadest spectrum was observed for *Desulfobacterales*, whose transcripts included also cbb3-type cytochrome c oxidase (ccoNO). *Desulfobacterales* transcribed most of the ROS defence genes at higher levels in ODB sediments than in FB sediments (Fig. S8).

**Discussion**

Sandy sediments in shallow coastal oceans are increasingly recognized as biocatalytic filters that harbour highly active microbial communities [51, 52]. Despite low concentrations of solutes and low organic carbon accumulation, high nutrient fluxes and oxygen uptake rates have been reported for sandy organic-poor sediments of temperate coastal seas [53, 54]. Recent studies led to a growing recognition of permeable coastal sediments in nitrogen removal by denitrification [55, 56]. Sulfate-reducing communities in sandy sediments received less attention, as permeable sediments were generally not considered a relevant habitat for this functional guild. Our results suggest that the extensive sandy areas may contribute a larger fraction to sulfate reduction in coastal oceans than currently assumed. High levels of sulfate reduction in the sandy sediments of the southern Baltic Sea were strongly supported by a novel order-level lineage of *Thermodesulfovibrionia*, revealing that the role of hitherto unrecognized specialists adapted to the conditions in permeable sediments may have been underestimated.

**Communities in organic-poor Odra Bank sediments sustain substantial sulfate reduction rates**

Despite strong differences in the concentrations of organic carbon, metals and sulfur compounds, similar SRR were measured in the top 20 cm of ODB and FB, emphasizing the importance of terminal electron acceptors other than oxygen for element cycling also in permeable sediments with advective pore water exchange. SRR at both sites were within the range of the few values that were previously reported for these areas [57, 58]. The rates are comparable to those in organic-rich muddy sediments in the Bay of Mecklenburg and Aarhus Bay (both in the Baltic Sea) [57, 59] and overlap with the rates measured in sediments in the North Sea [60, 61].

At ODB, the high permeability of the sandy sediments, associated with low concentrations of metals and organic matter, probably led to the intensive re-oxidation of biogenic sulfide and a lower accumulation of iron sulfides, which was reflected in low sulfide concentrations in the pore water and low TS concentrations [57, 58]. In contrast, high concentrations of dissolved Fe^2+^ revealed a suboxic zone from 2 to 8 cmbsf at FB, where sulfide was scavenged by reactive iron and/or manganese minerals [59–62].

**Different compositions of active SRM in muddy and sandy sediments**

The taxonomic annotation of dsrAB in the metatranscriptomes revealed that sulfate reduction in the sediments of the southern Baltic Sea was predominantly sustained by only a few orders. *Desulfobacterales (*class *Desulfobacteria),* and in particular the genus Sva0081 sediment group, demonstrated versatility as generalists that thrive in both muddy and sandy sediments. The flexibility of Sva0081 evident from our study is consistent with the cosmopolitan distribution of this genus in cold and temperate marine sediments [14, 62]. Furthermore, similar gene expression patterns of *Desulfobacterales* at our geochemically distinct study sites align with largely constitutive proteomes expressed by members of this order in response to different substrates [63]. Likewise, the order *Desulfobulbales* (class *Desulfobacteria*) was active in sulfate reduction at both study sites, but with an altered composition at the family level that coincided with changes in the gene expression profiles. *Thermodesulfovibrionia* played a significant role in sulfate reduction exclusively in ODB sediments, suggesting that permeable sediments also provide niches for specialists that can only compete with metabolically flexible generalists in this environment.

**Thermodesulfovibrionia thrive in sandy sediments at Odra Bank**

Significant proportions of *Thermodesulfovibrionia* in seafloor habitats were so far only reported from submarine venting systems, where hydrothermal fluids provide redox-active fluids that sustain rich microbial communities [44, 45]. The members of *Thermodesulfovibrionia*, which significantly contributed to sulfate reduction rates in the sandy sediments at ODB, belong to a novel order-level lineage, for which we propose the name *Arenidesulfomonadales* ord. nov.. Both the most abundant ribotype and the most active genotype of this ODB cluster are part of well-supported monophyletic clades that they form together with gene sequences and genomes, respectively, which cannot be assigned to any of the *Thermodesulfovibrionia* orders in GTDB and SILVA. A comprehensive screening of publicly available genome catalogues revealed two MAGs that are closely related to the MAG EMB267_AVHV_4_191 that represents the type species *Arenidesulfomonas baltica* sp. nov. of the ODB lineage named *Arenidesulfomonadales* ord. nov.. These MAGs originate from northern Barataria Bay (Mississippi River estuary), where mesohaline conditions of 5-15 psu are similar to the seawater salinity in the ODB region [64]. Likewise, the environmental 16S rRNA gene sequences that showed high similarity to the dominant *Thermodesulfovibrionia* 16S rRNA genes in our study originate from mesohaline sediments (Mariager Fjord, Baltic Sea). Hence, this clustering of 16S rRNA gene sequences and MAGs from oligohaline environments hint at salinity as an important environmental factor selecting for this *Thermodesulfovibrionia* lineage.

**Metabolic strategies of active SRM in muddy and sandy sediments**

Sulfate reduction in marine sediments has been largely attributed to *Desulfobacterota* and, therefore, considered primarily heterotrophic [1, 65]. However, both the gene expression and the gene inventory of *Thermodesulfovibrionia* suggest that hydrogenotrophic sulfate reduction may play a more important role in sandy, organic-poor sediments than in organic-rich sediments. In line with previous studies, functional annotations of transcripts imply a predominately heterotrophic metabolism of *Desulfobacterales* and *Desulfobulbales* (both phylum *Desulfobacterota*) in the Baltic Sea sediments that is based on the oxidation of primary fermentation products [1, 65]. The gene expression pattern of *Thermodesulfovibrionia* showed two essential differences from *Desulfobacterota* lineages in terms of carbon utilization and energy production, indicating a fundamentally different lifestyle. First, *Thermodesulfovibrionia* transcripts, as well as the MAG representing *Arenidesulfomonas baltica* sp. nov., lack major enzymes required for the oxidation of primary fermentation products. The second major difference was a 200-fold higher proportion of [NiFe] hydrogenases in *Thermodesulfovibrionia* transcripts, implying a higher investment in the utilization of hydrogen as an electron donor. Hydrogenase transcripts assigned to *Thermodesulfovibrionia* were dominated by [NiFe]-hydrogenase group 1a, which provides electron input for sulfate reduction. Notably, about one third of the *Thermodesulfovibrionia* hydrogenase transcripts were assigned to group 1d, an oxygen-tolerant group of [NiFe]-hydrogenases that was present only in negligible proportions in the transcript pool of *Desulfobacteria.* Oxygen tolerance of hydrogenases may facilitate the utilization of hydrogen in the sandy sediments with fluctuating oxygen concentrations.

Hydrogenotrophic sulfate reduction as mode of energy production was also proposed for *Thermodesulfovibrionia* from the Lost City hydrothermal field and from the Costa Rican convergent margin [44, 45]. It is noteworthy, that the chimney fluids contain millimolar hydrogen concentrations produced by reactions between water and certain minerals like serpentinization [44, 45, 66], whereas the major source of molecular hydrogen in sediments is most likely fermentation [67]. Fermentation-derived hydrogen can accumulate in the porewater of permeable, sandy sediments at nanomolar concentrations, a process facilitated by frequent shifts between oxic and anoxic conditions [68–70]. Therefore, both the uptake of hydrogen from the environment and a potential interspecies hydrogen transfer from syntrophic partners are possible mechanisms that may contribute to the hydrogen supply to *Thermodesulfovibrionia* in the Odra Bank sediments. In any case, hydrogenotrophy renders sulfate reduction by *Thermodesulfovibrionia* independent of the carbon products of fermentation, though not from fermentation itself. As a common intermediate produced by many fermentative processes, hydrogen may be more reliably available than certain carbon compounds produced by specific pathways.

The gene expression profile of *Thermodesulfovibrionia* at ODB suggests that hydrogenotrophic sulfate reduction was likely coupled to autotrophic carbon fixation via the Wood-Ljundahl pathway. The lack of key enzymes for the utilization of primary fermentation products implies that the bidirectional Wood-Ljungdahl pathway was used for autotrophic carbon fixation and not for the oxidation of organic carbon compounds in *Thermodesulfovibrionia*. A predominantly autotrophic carbon acquisition via the Wood-Ljungdahl pathway was also suggested for *Thermodesulfovibrionia* at the Costa Rican convergent margin as well as in aquifers and sediments of freshwater lakes [44, 45, 71, 72]. Hence, the fixation of CO_2_ in the organic-poor sediments is likely another important adaptation that enables the successful competition of *Thermodesulfovibrionia* with *Desulfobacterota* lineages at ODB. Autotrophic carbon fixation in *Thermodesulfovibrionia* implies that dissimilatory sulfate reduction in the sandy ODB sediments was less tightly coupled to carbon remineralization than in the muddy sediments of FB, where heterotrophic sulfate reduction by *Desulfobacteria* prevailed. However, hydrogenotrophy does not necessarily reduce the overall rate of carbon remineralization, as low hydrogen concentrations maintained by hydrogen-consuming taxa are an important prerequisite for fermentation processes to be energetically feasible [73, 74]. Hence, hydrogenotrophic sulfate reduction by *Thermodesulfovibrionia* may in turn promote carbon degradation, especially in case of complex compounds, by syntrophic partners and potentially other functional guilds in the sediment prokaryotic communities [75, 76].

In conclusion, our study shows that unrecognized SRM adapted to permeable sediments can contribute significantly to sulfate reduction in coastal oceans. In the case of *Thermodesulfovibrionia*, the decoupling of sulfate reduction and biomass production from the oxidation of organic compounds shows that the metabolism of such unrecognized taxa can lead to functional shifts in sulfate-reducing communities. Therefore, our results underscore the need to combine phylogenetic analyses of important functional groups such as SRM with environmental studies, including metatranscriptomics and ideally single-cell activity measurements (e.g., NanoSIMS or MAR-FISH), to deepen our understanding of their role in natural communities across different habitats.

**Data Availability**

Sequencing data were deposited in the European Nucleotide Archive using the GFBio brokerage service [77]. The V4-V5 16S rRNA amplicon sequencing reads, full-length 16S rRNA gene sequencing reads and metatranscriptomic reads are available under the study accession number PRJEB100525. All *Thermodesulfovibrionia* MAGs from the ODB sediments are available in [49]. Furthermore, the assembly of the MAG EMB267_AVHV_4_191 was archived under the accession number GCA_985800395. The latter genome assembly serves as the nomenclatural type of *Arenidesulfomonas baltica* sp. nov., the type species of the lineage consisting of *Arenidesulfomonadales* ord. nov., *Arenidesulfomonadaceae* fam. nov. and *Arenidesulfomonas* gen. nov., registered in SeqCode [78] (https://registry.seqco.de/registers/r:_yq8wyy8). The metagenomic raw reads are publicly available under PRJEB114799. All data on chemical measurements and SRR are included in Tab. S1.

**Funding**

This research was funded by the German Federal Ministry of Research, Technology and Space (Research grants 03F0848A and 03F0937A for the project MGF Baltic Sea) in the research mission sustainMare of the German Marine Research Alliance.

**Acknowledgements**

We thank Simone Bernsee, Christian Meeske and Ines Scherff for assistance during analysis of the sediment samples. We are also grateful to the master and crew of RV Alkor, the scientific cruise leader Stefan Sommer and the shipboard science party for their invaluable support during research cruise AL570. We thank the reviewers and the editorial team for their constructive comments and suggestions, which helped to improve the manuscript. We also acknowledge the SeqCode team for their support throughout the registration process.

**References**

1.	Jørgensen BB, Findlay AJ, Pellerin A. The biogeochemical sulfur cycle of marine sediments. *Front Microbiol* 2019;**10**:1–27. https://doi.org/10.3389/fmicb.2019.00849

2.	Thomsen U et al. Pathways of organic carbon oxidation in a deep lacustrine sediment, Lake Michigan. *Limnol Oceanogr* 2004;**49**:2046–2057. https://doi.org/10.4319/lo.2004.49.6.2046

3.	Pester M et al. Sulfate-reducing microorganisms in wetlands - fameless actors in carbon cycling and climate change. *Front Microbiol* 2012;**3**:1–19. https://doi.org/10.3389/fmicb.2012.00072

4.	Liesack W, Schnell S, Revsbech NP. Microbiology of flooded rice paddies. *FEMS Microbiol Rev* 2000;**24**:625–645. https://doi.org/10.1016/S0168-6445(00)00050-4

5.	Waite DW et al. Proposal to reclassify the proteobacterial classes deltaproteobacteria and oligoflexia, and the phylum thermodesulfobacteria into four phyla reflecting major functional capabilities. *Int J Syst Evol Microbiol* 2020;**70**:5972–6016. https://doi.org/10.1099/ijsem.0.004213

6.	Rabus R et al. A Post-Genomic View of the Ecophysiology, Catabolism and Biotechnological Relevance of Sulphate-Reducing Prokaryotes. In: Poole R (ed.), Advances in Microbial Physiology. Academic Press, 2015, 55–321.

7.	Diao M et al. Global diversity and inferred ecophysiology of microorganisms with the potential for dissimilatory sulfate/sulfite reduction. *FEMS Microbiol Rev* 2023;**47**:1–18. https://doi.org/10.1093/femsre/fuad058

8.	Middelburg JJ et al. Organic matter mineralization in marine systems. *Glob Planet Change* 1993;**8**:47–58. https://doi.org/10.1016/0921-8181(93)90062-S

9.	Gattuso JP, Frankignoulle M, Wollast R. Carbon and carbonate metabolism in coastal aquatic ecosystems. *Annu Rev Ecol Syst* 1998;**29**:405–434. https://doi.org/10.1146/annurev.ecolsys.29.1.405

10.	Jørgensen BB. Mineralisation of organic matter in the sea bed-the role of sulphate reduction. *Nature* 1982;**296**:643–645.

11.	Mußmann M et al. Diversity and vertical distribution of cultured and uncultured Deltaproteobacteria in an intertidal mud flat of the Wadden Sea. *Environ Microbiol* 2005;**7**:405–418. https://doi.org/10.1111/j.1462-2920.2005.00708.x

12.	Leloup J et al. Sulfate-reducing bacteria in marine sediment (Aarhus Bay, Denmark): Abundance and diversity related to geochemical zonation. *Environ Microbiol* 2009;**11**:1278–1291. https://doi.org/10.1111/j.1462-2920.2008.01855.x

13.	Petro C et al. Marine deep biosphere microbial communities assemble in near-surface sediments in Aarhus bay. *Front Microbiol* 2019;**10**:1–13. https://doi.org/10.3389/fmicb.2019.00758

14.	Fonseca A et al. Bacterial community of sediments under the Eastern Boundary Current System shows high microdiversity and a latitudinal spatial pattern. *Front Microbiol* 2022;**13**:1–13. https://doi.org/10.3389/fmicb.2022.1016418

15.	Perner M et al. Environmental changes affect the microbial release of hydrogen sulfide and methane from sediments at Boknis Eck (SW Baltic Sea). *Front Microbiol* 2022;**13**:1–13. https://doi.org/10.3389/fmicb.2022.1096062

16.	Reyes C et al. Potentially Active Iron, Sulfur, and Sulfate Reducing Bacteria in Skagerrak and Bothnian Bay Sediments. *Geomicrobiol J* 2017;**34**:840–850. https://doi.org/10.1080/01490451.2017.1281360

17.	Hall SJ. The continental shelf benthic ecosystem: current status, agents for change and future prospects. *Environ Conserv* 2002;**29**:350–374. https://doi.org/10.1017/S0376892902000243

18.	Wilson RJ et al. A synthetic map of the north-west European Shelf sedimentary environment for applications in marine science. *Earth Syst Sci Data* 2018;**10**:109–130. https://doi.org/10.5194/essd-10-109-2018

19.	Jørgensen BB. Sulfate reduction in marine sediments from the baltic sea-north sea transition. *Ophelia* 1989;**31**:1–15. https://doi.org/10.1080/00785326.1989.10430847

20.	Upton AC et al. Seasonal benthic microbial activity in the southern North Sea - Oxygen uptake and sulphate reduction. *Mar Ecol Prog Ser* 1993;**101**:273–282. https://doi.org/10.3354/meps101273

21.	Dolla A, Fournier M, Dermoun Z. Oxygen defense in sulfate-reducing bacteria. *J Biotechnol* 2006;**126**:87–100. https://doi.org/10.1016/j.jbiotec.2006.03.041

22.	Dyksma S, Pester M. Growth of sulfate-reducing Desulfobacterota and Bacillota at periodic oxygen stress of 50% air-O2 saturation. *Microbiome* 2024;**12**. https://doi.org/10.1186/s40168-024-01909-7

23.	Chen YJ et al. Hydrodynamic disturbance controls microbial community assembly and biogeochemical processes in coastal sediments. *ISME J* 2022;**16**:750–763. https://doi.org/10.1038/s41396-021-01111-9

24.	Ehlert von Ahn CM et al. A Multi-Tracer Study of Fresh Water Sources for a Temperate Urbanized Coastal Bay (Southern Baltic Sea). *Front Environ Sci* 2021;**9**:1–20. https://doi.org/10.3389/fenvs.2021.642346

25.	Cline JD. Spectrophotometric Determination of Hydrogen Sulfide in Natural Waters1. *Limnol Oceanogr* 1969;**14**:454–458. https://doi.org/10.4319/lo.1969.14.3.0454

26.	Jørgensen BB. A comparison of methods for the quantification of bacterial sulfate reduction in coastal marine sediments: I. Measurement with radiotracer techniques. *Geomicrobiol J* 1978;**1**:11–27. https://doi.org/10.1080/01490457809377721

27.	Kallmeyer J et al. A cold chromium distillation procedure for radiolabeled sulfide applied to sulfate reduction measurements. *Limnol Oceanogr Methods* 2004;**2**:171–180. https://doi.org/10.4319/lom.2004.2.171

28.	Bushnell B. BBMap: short-read aligner, and other bioinformatics tools. 2015.

29.	Hyatt D et al. Integrated nr Database in Protein Annotation System and Its Localization. *Nat Commun* 2010;**6**:1–8.

30.	Buchfink B, Reuter K, Drost HG. Sensitive protein alignments at tree-of-life scale using DIAMOND. *Nat Methods* 2021;**18**:366–368. https://doi.org/10.1038/s41592-021-01101-x

31.	Kanehisa M et al. KEGG as a reference resource for gene and protein annotation. *Nucleic Acids Res* 2016;**44**:D457–D462. https://doi.org/10.1093/nar/gkv1070

32.	Rasko DA, Myers GSA, Ravel J. Visualization of comparative genomic analyses by BLAST score ratio. *BMC Bioinformatics* 2005;**6**:1–7. https://doi.org/10.1186/1471-2105-6-2

33.	Eddy SR. Profile hidden Markov models. *Bioinformatics* 1998;**14**:755–763. https://doi.org/10.1093/bioinformatics/14.9.755

34.	Pelikan C et al. Glacial Runoff Promotes Deep Burial of Sulfur Cycling-Associated Microorganisms in Marine Sediments. *Front Microbiol* 2019;**10**:1–17. https://doi.org/10.3389/fmicb.2019.02558

35.	Parks DH et al. A standardized bacterial taxonomy based on genome phylogeny substantially revises the tree of life. *Nat Biotechnol* 2018;**36**:996. https://doi.org/10.1038/nbt.4229

36.	Von Meijenfeldt FAB et al. Robust taxonomic classification of uncharted microbial sequences and bins with CAT and BAT. *Genome Biol* 2019;**20**:1–14. https://doi.org/10.1186/s13059-019-1817-x

37.	Langmead B, Salzberg SL. Fast gapped-read alignment with Bowtie 2. *Nat Methods* 2012;**9**:357–359. https://doi.org/10.1038/nmeth.1923

38.	Liao Y, Smyth GK, Shi W. FeatureCounts: An efficient general purpose program for assigning sequence reads to genomic features. *Bioinformatics* 2014;**30**:923–930. https://doi.org/10.1093/bioinformatics/btt656

39.	Lane D. 16S/23S rRNA Sequencing. In: Stackebrandt E, Goodfellow M (eds.), Nucleic Acid Techniques in Bacterial Systematics. John Wiley and Sons, 1991, 115–175.

40.	Parada AE, Needham DM, Fuhrman JA. Every base matters: Assessing small subunit rRNA primers for marine microbiomes with mock communities, time series and global field samples. *Environ Microbiol* 2016;**18**:1403–1414. https://doi.org/10.1111/1462-2920.13023

41.	Callahan BJ et al. DADA2: High-resolution sample inference from Illumina amplicon data. *Nat Methods* 2016;**13**:581–583. https://doi.org/10.1038/nmeth.3869

42.	Martin M. Cutadapt removes adapter sequences from high-throughput sequencing reads. *EMBnet J* 2011;**17**:10–12. https://doi.org/https://doi.org/10.14806/ej.17.1.200

43.	Quast C et al. The SILVA ribosomal RNA gene database project: Improved data processing and web-based tools. *Nucleic Acids Res* 2013;**41**:590–596. https://doi.org/10.1093/nar/gks1219

44.	Brazelton WJ et al. Metabolic Strategies Shared by Basement Residents of the Lost City Hydrothermal Field. *Appl Environ Microbiol* 2022;**88**. https://doi.org/10.1128/aem.00929-22

45.	Rogers TJ et al. Chemolithoautotroph distributions across the subsurface of a convergent margin. *ISME J* 2023;**17**:140–150. https://doi.org/10.1038/s41396-022-01331-7

46.	Pruesse E, Peplies J, Glöckner FO. SINA: Accurate high-throughput multiple sequence alignment of ribosomal RNA genes. *Bioinformatics* 2012;**28**:1823–1829. https://doi.org/10.1093/bioinformatics/bts252

47.	Price MN, Dehal PS, Arkin AP. FastTree 2 - Approximately maximum-likelihood trees for large alignments. *PLoS One* 2010;**5**. https://doi.org/10.1371/journal.pone.0009490

48.	Letunic I, Bork P. Interactive Tree Of Life (iTOL): An online tool for phylogenetic tree display and annotation. *Bioinformatics* 2007;**23**:127–128. https://doi.org/10.1093/bioinformatics/btl529

49.	Piontek J et al. Metagenome-assembled genomes from Odra Bank sediments (2025). https://doi.org/10.12754/data-2025-0013

50.	Greening C et al. Genomic and metagenomic surveys of hydrogenase distribution indicate H 2 is a widely utilised energy source for microbial growth and survival. *ISME J* 2016;**10**:761–777. https://doi.org/10.1038/ismej.2015.153

51.	Huettel M, Berg P, Kostka JE. Benthic exchange and biogeochemical cycling in permeable sediments. *Ann Rev Mar Sci* 2014;**6**:23–51. https://doi.org/10.1146/annurev-marine-051413-012706

52.	Probandt D et al. Microbial life on a sand grain: From bulk sediment to single grains. *ISME J* 2018;**12**:623–633. https://doi.org/10.1038/ismej.2017.197

53.	Janssen F, Huettel M, Witte U. Pore-water advection and solute fluxes in permeable marine sediments (II): Benthic respiration at three sandy sites with different permeabilities (German Bight, North Sea). *Limnol Oceanogr* 2005;**50**:779–792. https://doi.org/10.4319/lo.2005.50.3.0779

54.	Rusch A et al. Benthic oxygen consumption and organic matter turnover in organic-poor, permeable shelf sands. *Aquat Geochemistry* 2006;**12**:1–19. https://doi.org/10.1007/s10498-005-0784-x

55.	Jalaluddin FM et al. Microenvironments on individual sand grains enhance nitrogen loss in coastal sediments. *Sci Rep* 2025;**15**:1–14. https://doi.org/10.1038/s41598-025-00755-3

56.	Marchant HK et al. Denitrifying community in coastal sediments performs aerobic and anaerobic respiration simultaneously. *ISME J* 2017;**11**:1799–1812. https://doi.org/10.1038/ismej.2017.51

57.	Lipka M et al. Solute reservoirs reflect variability of early diagenetic processes in temperate brackish surface sediments. *Front Mar Sci* 2018;**9**:1–20. https://doi.org/10.3389/fmars.2018.00413

58.	Rooze J et al. Bottom-trawling signals lost in sediment: A combined biogeochemical and modeling approach to early diagenesis in a perturbed coastal area of the southern Baltic Sea. *Sci Total Environ* 2024;**906**. https://doi.org/10.1016/j.scitotenv.2023.167551

59.	Jørgensen BB et al. Organoclastic sulfate reduction in the sulfate-methane transition of marine sediments. *Geochim Cosmochim Acta* 2019;**254**:231–245. https://doi.org/10.1016/j.gca.2019.03.016

60.	Böttcher ME et al. The biogeochemistry, stable isotope geochemistry, and microbial community structure of a temperate intertidal mudflat: An integrated study. *Cont Shelf Res* 2000;**20**:1749–1769. https://doi.org/10.1016/S0278-4343(00)00046-7

61.	Al-Raei AM et al. Seasonal dynamics of microbial sulfate reduction in temperate intertidal surface sediments: controls by temperature and organic matter. *Ocean Dyn* 2009;**59**:351–370. https://doi.org/10.1007/s10236-009-0186-5

62.	Dyksma S et al. Evidence for H2 consumption by uncultured Desulfobacterales in coastal sediments. *Environ Microbiol* 2018;**20**:450–461. https://doi.org/10.1111/1462-2920.13880

63.	Wöhlbrand L et al. Key role of Desulfobacteraceae in C/S cycles of marine sediments is based on congeneric catabolic-regulatory networks. *Sci Adv* 2025;**11**:1–16. https://doi.org/10.1126/sciadv.ads5631

64.	Bianchi TS et al. Impacts of diverted freshwater on dissolved organic matter and microbial communities in Barataria Bay, Louisiana, U.S.A. *Mar Environ Res* 2011;**72**:248–257. https://doi.org/10.1016/j.marenvres.2011.09.007

65.	Muyzer G, Stams AJM. The ecology and biotechnology of sulphate-reducing bacteria. *Nat Rev Microbiol* 2008;**6**:441–454. https://doi.org/10.1038/nrmicro1892

66.	Kelley DS et al. An off-axis hydrothermal vent field near the mid-atlantic ridge at 30° n. *Nature* 2001;**412**:145–149. https://doi.org/10.1038/35084000

67.	Laanbroek H, Veldkamp H. Microbial interactions in sediment communities. *Philos Trans R Soc B* 1982;**297**:533–550. https://doi.org/10.1098/rstb.1982.0059

68.	Kessler AJ et al. Bacterial fermentation and respiration processes are uncoupled in anoxic permeable sediments. *Nat Microbiol* 2019;**4**:1014–1023. https://doi.org/10.1038/s41564-019-0391-z

69.	Nauer PA et al. Pulses of labile carbon cause transient decoupling of fermentation and respiration in permeable sediments. *Limnol Oceanogr* 2023;**68**:2141–2152. https://doi.org/10.1002/lno.12411

70.	Bourke MF et al. Metabolism in anoxic permeable sediments is dominated by eukaryotic dark fermentation. *Nat Geosci* 2017;**10**:30–35. https://doi.org/10.1038/ngeo2843

71.	Mosley OE, Gios E, Handley KM. Implications for nitrogen and sulphur cycles: Phylogeny and niche-range of Nitrospirota in terrestrial aquifers. *ISME Commun* 2024;**4**. https://doi.org/10.1093/ismeco/ycae047

72.	Ruiz-Blas F et al. Metabolic features that select for Bathyarchaeia in modern ferruginous lacustrine subsurface sediments. *ISME Commun* 2024;**4**. https://doi.org/10.1093/ismeco/ycae112

73.	Lee MJ, Zinder SH. Hydrogen Partial Pressures in a Thermophilic Acetate-Oxidizing Methanogenic Coculture. *Appl Environ Microbiol* 1988;**54**:1457–1461. https://doi.org/10.1128/aem.54.6.1457-1461.1988

74.	Birg A et al. Hydrogen Availability Is Dependent on the Actions of Both Hydrogen-Producing and Hydrogen-Consuming Microbes. *Dig Dis Sci* 2023;**68**:1253–1259. https://doi.org/10.1007/s10620-022-07743-x

75.	Wallrabenstein C, Hauschild E, Schink B. Pure culture and cytological properties of ‘Syntriphobacter wolini’. *FEMS Microbiol Lett* 1994;**123**:249–254. https://doi.org/10.1111/j.1574-6968.1994.tb07232.x

76.	Ozuolmez D et al. Methanogenic archaea and sulfate reducing bacteria co-cultured on acetate: Teamwork or coexistence? *Front Microbiol* 2015;**6**:1–12. https://doi.org/10.3389/fmicb.2015.00492

77.	Diepenbroek M et al. Towards an Integrated Biodiversity and Ecological Research Data Management and Archiving Platform : The German federation for the curation of biological data (GFBio). *Informatik* 2014;1711–1721.

78.	Hedlund BP et al. SeqCode: a nomenclatural code for prokaryotes described from sequence data. *Nat Microbiol* 2022;**7**:1702–1708. https://doi.org/10.1038/s41564-022-01214-9

79.	E.U. Copernicus Marine Service Information (CMEMS). Marine Data Store (MDS). Global Ocean Biogeochemistry Analysis and Forecast. doi.org/10.48670/moi-00015.

**Figure Captions**

**Fig. 1.** Location of the Fehmarn Belt and Odra Bank sampling sites (A) and the seasonal development of chlorophyll-a concentrations in the study areas in the year 2022 (B). The time slot highlighted in grey in panel B indicates the time of sampling for metatranscriptomes, 16S rRNA amplicon sequences and geochemical analyses. Chlorophyll *a* concentrations were derived from the Copernicus product Global Ocean Biogeochemistry Analysis and Forecast [79].

ALT TEXT FIG. 1. Map showing the sampling sites Fehmarn Belt and Odra Bank in the Baltic Sea (left) and a graph visualizing the seasonal trend of chlorophyll-a concentration in the regions (right).

**Fig. 2**. Depth profiles of total organic carbon (TOC) and total sulfur (TS) concentrations, porewater constituents, sulfate reduction rates (SRR) and proportions of *dsrAB* transcripts in Fehmarn Belt (TPM, transcripts per million) (stations 9, 11, 33) and Odra Bank sediments (stations 44, 53, 62). Please note the different axis scales for the Fehmarn Belt and Odra Bank stations.

ALT TEXT FIG. 2. Graphs comparing total organic carbon, total sulphur, different porewater constituents, sulfate reduction rates and proportions of *dsrAB* transcripts at Fehmarn Belt and Odra Bank.

**Fig. 3.** Taxonomic assignments of *dsrA* (left panels) and *dsrB* transcripts (right panels) at order level for Fehmarn Belt (A) and Odra Bank (B) sediments at 0-1 centimeters below seafloor (cmbsf), 3-4 cmbsf and 6-7 cmbsf. Respective phyla are given in brackets. Orders with average proportions of <1% of dsrA in both study areas were summarized in the category “other orders” (see Tab. S2 for transcript proportions of all *dsrAB*-transcribing lineages).

ALT TEXT FIG. 3. Stacked bar plots comparing the taxonomic affiliation of *dsrA* and *dsrB* transcripts at Fehmarn Belt and Odra Bank.

**Fig. 4.** Taxonomic composition of *Desulfobacterales* (left panels) and *Desulfobulbales* (right panels) at Fehmarn Belt (A) and Odra Bank (B) derived from 16S rRNA sequences.

ALT TEXT FIG. 4. Stacked bar plots showing the taxonomic composition of *Desulfobacterales* and *Desulfobulbales* at genus level, based on 16S rRNA sequences.

**Fig. 5.** Phylogenetic analysis of *Thermodesulfovibrionia* in Odra Bank sediments. The phylogenetic tree includes full-length 16S rRNA gene sequences from Odra Bank samples as well as reference sequences obtained from GTDB genomes (r232) and environmental sequences from the SILVA collection. Symbols denote the habitats of the environmental sequences.

ALT TEXT FIG. 5. Phylogenetic tree of *Thermodesulfovibrionia* full-length 16S rRNA gene sequences in Odra Bank sediments, together with reference sequences from public databases.

**Fig. 6.** Taxonomic annotation of *Thermodesulfovibrionia* from Odra Bank sediments as inferred from the alignment of partial 16S rRNA sequences with the full-length 16S rRNA gene sequences shown in the phylogenetic tree in Fig. 5. The proportion of sequences that did not align to a full-length 16S rRNA gene sequence are referred to as “not assigned”.

ALT TEXT FIG. 6. Stacked bar plot showing the taxonomic composition of *Thermodesulfovibrionia* in Odra Bank sediments as inferred from the alignment of partial 16S rRNA sequences with the full-length 16S rRNA gene sequences.

**Fig. 7.** Differences in gene expression patterns of major sulfate-reducing lineages at Fehmarn Belt (FB) and Odra Bank (ODB) based on KEGG ortholog annotations of transcripts. The non-metric multi-dimensional scaling plot is based on Bray-Curtis dissimilarities between the KEGG ortholog composition of the transcript pools (stress = 0.08).

ALT TEXT FIG. 7. Non-metric multi-dimensional scaling plot showing the differences in gene expression patterns of major sulfate-reducing lineages at Fehmarn Belt and Odra Bank.

**Fig. 8.** Overview on transcripts of important functional genes involved in the carbon metabolism (A), energy metabolism (B, C) and defence against reactive oxygen species (D) of the most important sulfate-reducing lineages at Fehmarn Belt and Odra Bank. For abbreviations of the gene names and transcription levels of the gene see Tab. S4.

ALT TEXT FIG. 8. Graphical representation of transcripts of selected functional genes involved in carbon and energy metabolism and the defence against reactive oxygen species in the most important sulfate-reducing lineages in this study.
